# Supplementary material for: Eukaryotic domestication of a bacterial immune protein following horizontal transfer
Source: bioRxiv. 2026 May 22:2026.04.30.722052. Preprint. [Version 3] doi: 10.64898/2026.04.30.722052 (PMC13142454; doi:10.64898/2026.04.30.722052)
Supplement: 1 — Figure S1. TIR domain search across multiple PFAM HMMs A. Schematic of search strategy employed to find diverse TIR domains across the eukaryotic tree of life. B. Collector’s curve of searches (left) and C. the total number of hits per Pfam Hmm search. Figure S2. TIR domain clades are robust across alignment parameters Four phylogenetic trees generated by IQtree from varied alignments, showing clades supported across all trees and a schematic of the clade topologies. A. Alignment generated via MUSCLE with a 20% trim. B. Alignment generated via MUSCLE with a 10% trim. C. Alignment generated via MAFFT with a 20% trim. D. Alignment generated via Mafft with a 10% trim. E-H, Collapsed clades from the four IQtrees (E-Muscle 20% trim, F-Muscle 10% trim, G-MAFFT 20% trim, H-MAFFT 10% trim) with clades colored as in Fig. 1. Only nodes with 50 or greater bootstrap values are shown, unsupported nodes are collapsed to polytomies. Figure S3. TIRs family members in clades 1 & 4 A. A circular maximum likelihood phylogenetic tree generated by IQtree of TIR domains from Clade 1. The majority of sequences in this clade were Metazoan TIRs. Colored wedges designate protein clades: MyD88 (yellow), TIRAP (red), TLRs (blue), Interleukin receptors (dark teal). B. Maximum likelihood phylogenetic tree generated by IQtree of TIR domains from Clade 8. Clade 8 was a mainly bacterial clade into which a subset of Metazoan TIRs, notably SARM1, clustered. C. A circular maximum likelihood phylogenetic tree generated by IQtree of TIR domains from Clade 4. This clade contained all of the TIR domains from land plants (Embryophyta). White circles indicate notable TIR domain-containing proteins: (AB) Hs = Homo sapiens, (C) At = Arabidopsis thaliana, Al = Arabidopsis lyrata, Bd = Brachypodium distachyon, Nb = Nicotiana bethamiana. Both leaf color and strip color indicate the eukaryotic supergroup. Wedges represent collapsed parts of the phylogenetic trees. Trees are rooted on outgroup sequences (gray wedg [file NIHPP2026.04.30.722052V3-supplement-1.pdf]

bioRxiv preprint doi: https://doi.org/10.64898/2026.04.30.722052; this version posted May 22, 2026. The copyright holder for this preprint (which was not certified by peer review) is the author/funder, who has granted bioRxiv a license to display the preprint in perpetuity. It is made available under a CC-BY 4.0 International license.

Supplemental Figures, Tables, and Movies

Legend: Supp. Table. 1: **TIR domain containing proteins from phylogenetic analysis.** A table displaying information on all proteins in the phylogenetic analysis (Fig. 1). Data includes the sequence name, full protein amino acid sequence, the domains present, the clade in the phylogenetic tree of Fig. 1, taxonomic information, and which domain of life the sequence is from. Domain information is displayed as <Name of the sequence>, <total length of protein>, <start of domain> | <end of domain> | <domain name> | <domain Pfam ID>. Multiple domains are separated by a comma.

Legend: Supp. Table. 2: **Neighboring genes in the *tirB, tirC,* and *tirD* loci.** The neighboring genes (3 upstream, 3 downstream) around *tirB, tirC,* and *tirD* in the *Dictyostelium discoideum* AX2 genome are identified by the TIR locus, the name of the gene, the position of the gene relative to the nearby TIR gene, the gene annotation, and gene symbol. We searched both the genomic nucleotide sequences as well as the exonic sequences with nBLAST and display the top hit result in the table. Because we excluded all Dictyostelia, many of these genes yielded no BLAST hits (labeled as “No hit found”). We next translated all of these nucleotide sequences into amino acid sequences and used Hmmscan to determine what domains are present and used Jackhmmer to look for homology to other proteins. From Jackhmmer, we are reporting the taxonomic classification (at the domain level) of the significant hits. We then show the nucleotide sequences (both the genomic sequence and the exon-only sequence). Finally, we display the protein sequence.

Legend: Supp. Table. 3: **Strains used in Study**

Legend: Supp. Table. 4: **Plasmids used in Study**

Legend: Supp. Table. 5: **Primers used in Study**

Legend: **S6 File: TIR Atlas Tree, MUSCLE, 20%Trim**
Newick file of maximum likelihood phylogenetic tree of TIRs generated via MUSCLE alignment with TrimAL (-gt 0.2) with IQtree (v3.0.1, -m MFP, -bb 1000). Newick file is used in Figs 1 and Supp. Fig. 2. Node support calculated from ultrafast Bootstraps.

Legend: **S7 File: TIR Atlas Tree, MUSCLE, 10%Trim**
Newick file of maximum likelihood phylogenetic tree of TIRs generated via MUSCLE alignment with TrimAL (-gt 0.1) with IQtree (v3.0.1, -m MFP, -bb 1000). Newick file is used in Supp. Fig. 2. Node support calculated from ultrafast Bootstraps.

Legend: **S8 File: TIR Atlas Tree, MAFFT, 20%Trim**
Newick file of maximum likelihood phylogenetic tree of TIRs generated via MAFFT alignment with TrimAL (-gt 0.2) with IQtree (v3.0.1, -m MFP, -bb 1000). Newick file is used in Supp. Fig. 2. Node support calculated from ultrafast Bootstraps.

bioRxiv preprint doi: https://doi.org/10.64898/2026.04.30.722052; this version posted May 22, 2026. The copyright holder for this preprint (which was not certified by peer review) is the author/funder, who has granted bioRxiv a license to display the preprint in perpetuity. It is made available under a CC-BY 4.0 International license.

Legend: **S9 File: TIR Atlas Tree, MAFFT, 10%Trim**
Newick file of maximum likelihood phylogenetic tree of TIRs generated via MAFFT alignment with TrimAL (-gt 0.1) with IQtree (v3.0.1, -m MFP, -bb 1000). Newick file is used in Supp. Fig. 2. Node support calculated from ultrafast Bootstraps.

Legend: **S10 File: Clade 1 Tree**
Newick file of maximum likelihood phylogenetic tree of Clade 1 TIRs generated via MUSCLE alignment with TrimAL (-gt 0.05) with IQtree (v3.0.1, -m MFP, -bb 1000). Newick file is used in Supp. Fig. 3. Node support calculated from ultrafast Bootstraps.

Legend: **S11 File: Clade 4 Tree**
Newick file of maximum likelihood phylogenetic tree of Clade 4 TIRs generated via MUSCLE alignment with TrimAL (-gt 0.05) with IQtree (v3.0.1, -m MFP, -bb 1000). Newick file is used in Supp. Fig. 3. Node support calculated from ultrafast Bootstraps.

Legend: **S12 File: Clade 6 Tree**
Newick file of maximum likelihood phylogenetic tree of Clade 6 TIRs generated via MUSCLE alignment with TrimAL (-gt 0.05) with IQtree (v3.0.1, -m MFP, -bb 1000). Newick file is used in Figs 1. Node support calculated from ultrafast Bootstraps.

Legend: **S13 File: Clade 8 Tree**
Newick file of maximum likelihood phylogenetic tree of Clade 8 TIRs generated via MUSCLE alignment with TrimAL (-gt 0.05) with IQtree (v3.0.1, -m MFP, -bb 1000). Newick file is used in Supp. Fig. 3. Node support calculated from ultrafast Bootstraps.

Legend: Supp. Mov. 1: ***D. discoideum* expressing full length WT tirC**
Fluorescent microscopy time lapse of *D. discoideum* expressing full length WT tirC-mNeonGreen. The time lapse covers 16 hours with a 10 minute frame interval. The time stamp in the top right corner displays times in an hour:minute format.

Legend: Supp. Mov. 2: ***D. discoideum* expressing tirC ΔNT**
Fluorescent microscopy time lapse of D. discoideum expressing tirC ΔNT-mNeonGreen. The time lapse covers 18 hours with a 10 minute frame interval. The time stamp in the top right corner displays times in an hour:minute format. Boxes highlight mNeonGreen-positive cells undergoing cell lysis.

bioRxiv preprint doi: https://doi.org/10.64898/2026.04.30.722052; this version posted May 22, 2026. The copyright holder for this preprint (which was not certified by peer review) is the author/funder, who has granted bioRxiv a license to display the preprint in perpetuity. It is made available under a CC-BY 4.0 International license.

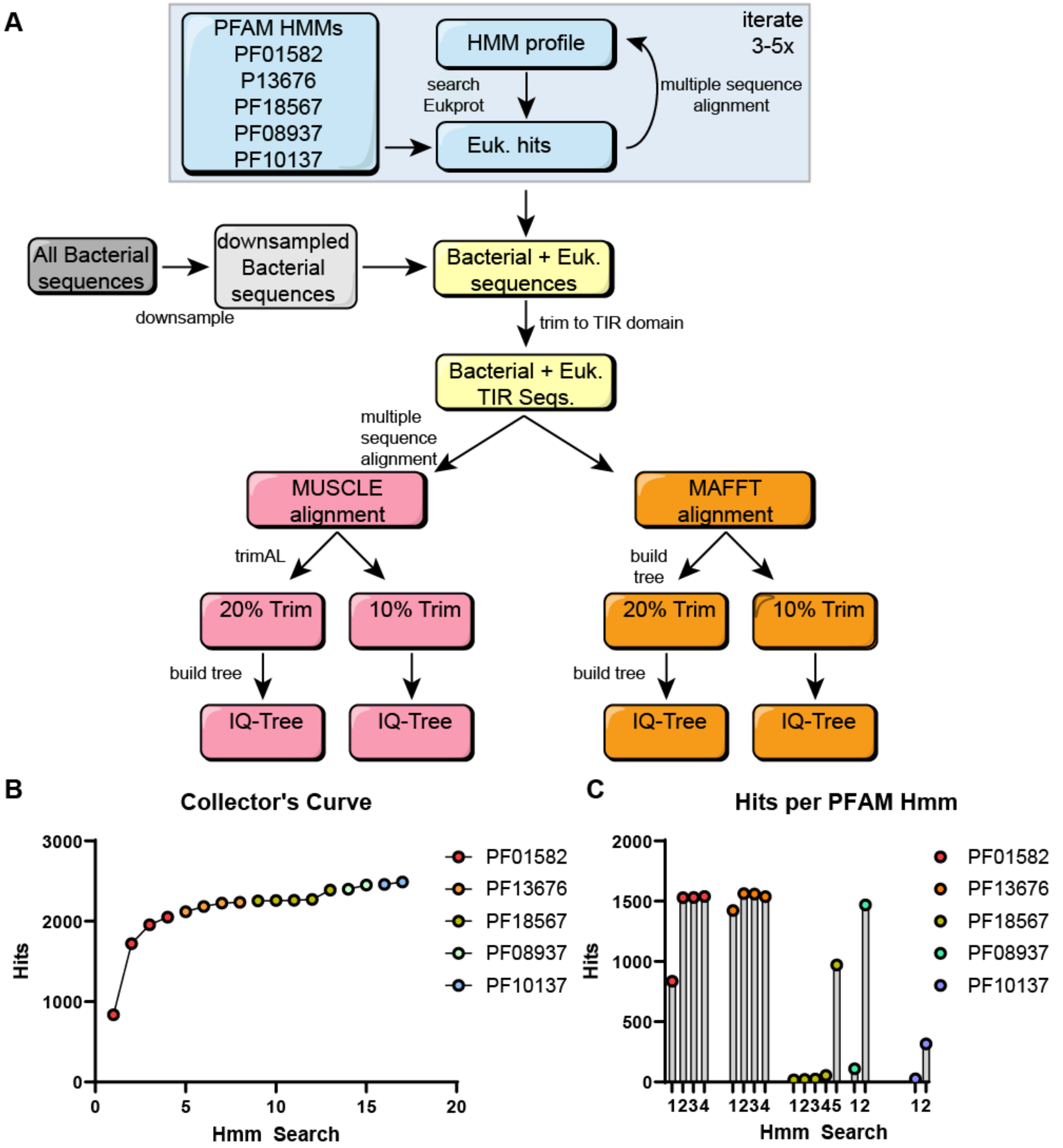

Figure S1. TIR domain search across multiple PFAM HMMs

A. Schematic of search strategy employed to find diverse TIR domains across the eukaryotic tree of life. **B.** Collector's curve of searches (left) and **C.** the total number of hits per Pfam Hmm search.

bioRxiv preprint doi: https://doi.org/10.64898/2026.04.30.722052; this version posted May 22, 2026. The copyright holder for this preprint (which was not certified by peer review) is the author/funder, who has granted bioRxiv a license to display the preprint in perpetuity. It is made available under a CC-BY 4.0 International license.

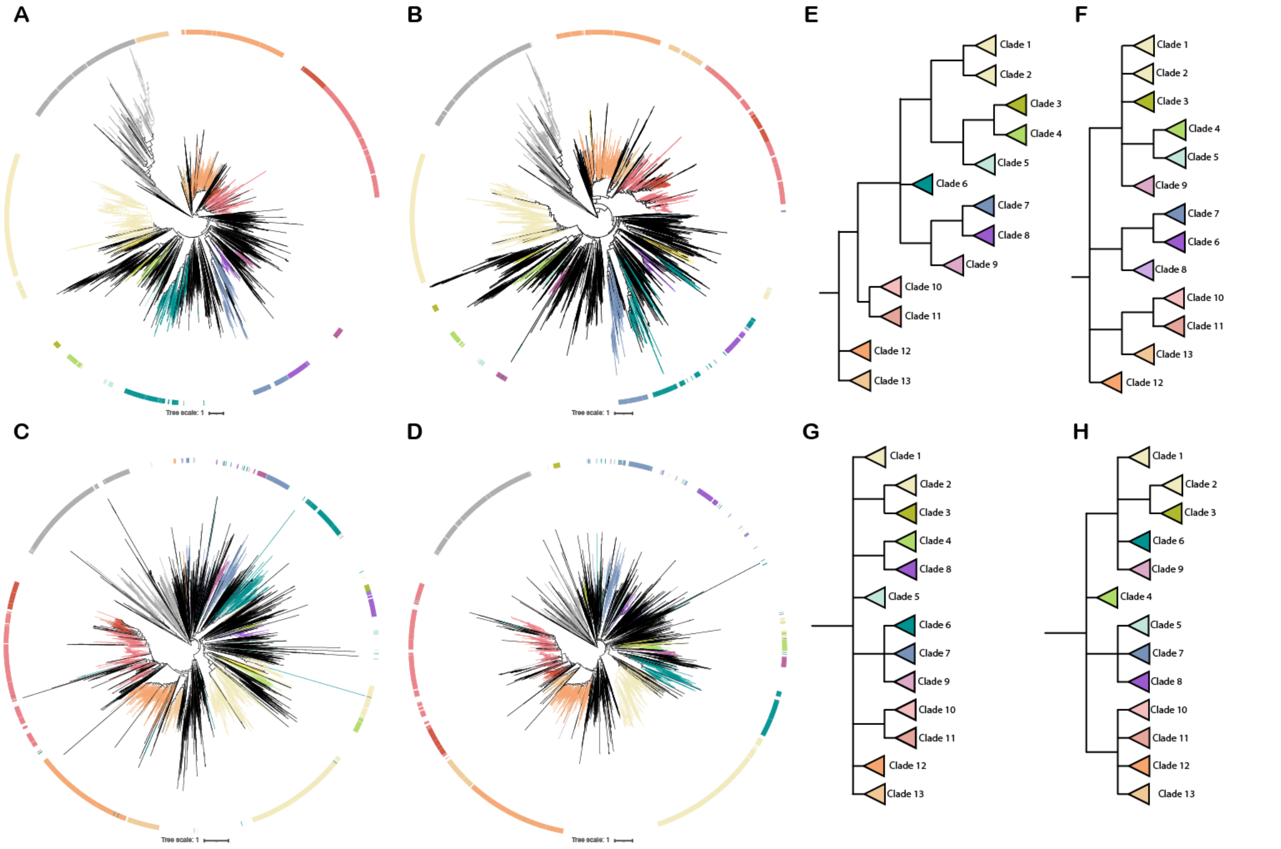

Figure S2. TIR domain clades are robust across alignment parameters
Four phylogenetic trees generated by IQtree from varied alignments, showing clades supported across all trees and a schematic of the clade topologies. **A.** Alignment generated via MUSCLE with a 20% trim. **B.** Alignment generated via MUSCLE with a 10% trim. **C.** Alignment generated via MAFFT with a 20% trim. **D.** Alignment generated via Mafft with a 10% trim. **E-H,** Collapsed clades from the four IQtrees (**E**-Muscle 20% trim, **F**-Muscle 10% trim, **G**-MAFFT 20% trim, **H**-MAFFT 10% trim) with clades colored as in Fig. 1. Only nodes with 50 or greater bootstrap values are shown, unsupported nodes are collapsed to polytomies.

bioRxiv preprint doi: https://doi.org/10.64898/2026.04.30.722052; this version posted May 22, 2026. The copyright holder for this preprint (which was not certified by peer review) is the author/funder, who has granted bioRxiv a license to display the preprint in perpetuity. It is made available under a CC-BY 4.0 International license.

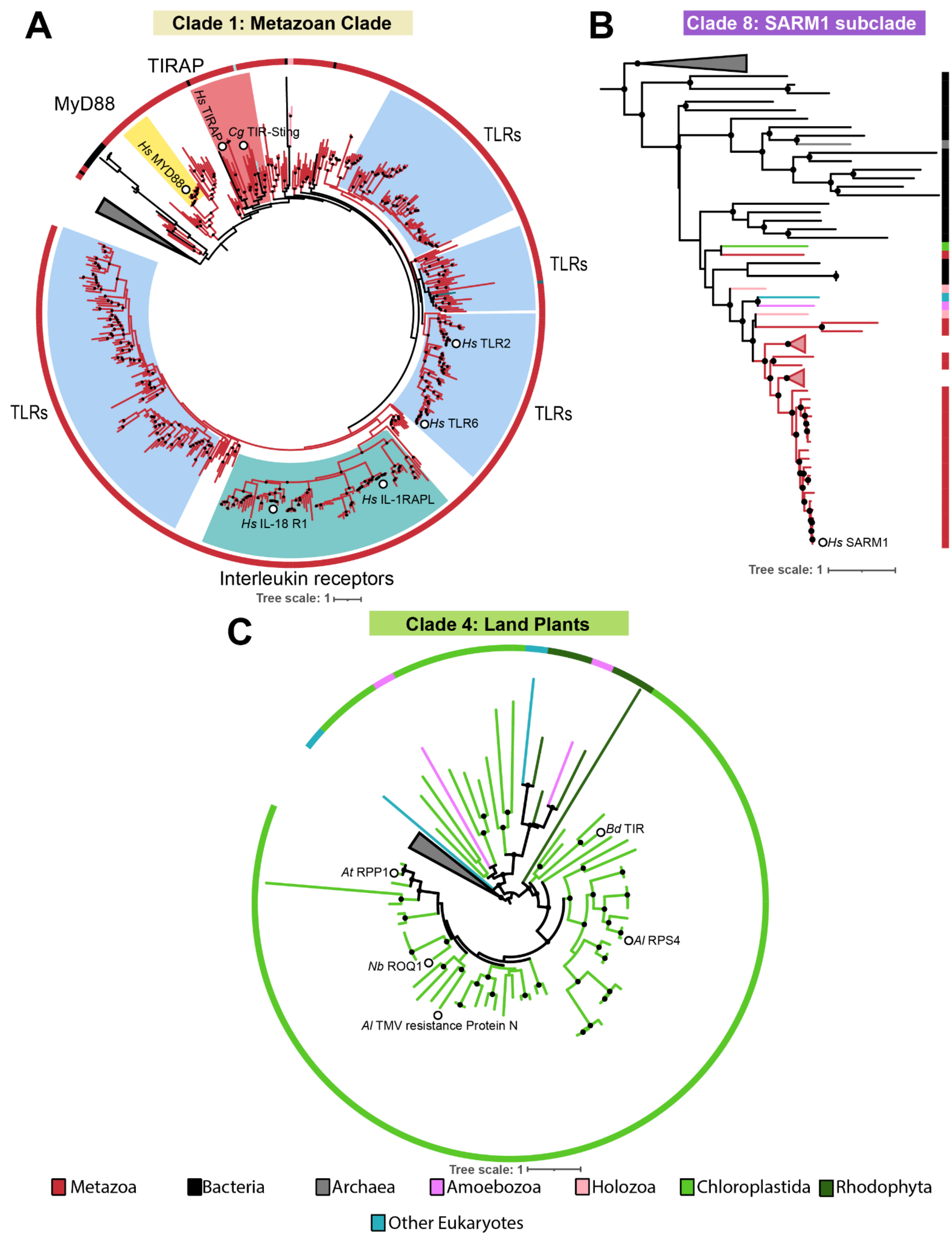

1150
1151 **Figure S3. TIRs family members in clades 1, 8, & 4**
1152 **A.** A circular maximum likelihood phylogenetic tree generated by IQtree of TIR domains from
1153 Clade 1. The majority of sequences in this clade were Metazoan TIRs. Colored wedges

bioRxiv preprint doi: https://doi.org/10.64898/2026.04.30.722052; this version posted May 22, 2026. The copyright holder for this preprint (which was not certified by peer review) is the author/funder, who has granted bioRxiv a license to display the preprint in perpetuity. It is made available under a CC-BY 4.0 International license.

designate protein clades: MyD88 (yellow), TIRAP (red), TLRs (blue), Interleukin receptors (dark teal). **B.** Maximum likelihood phylogenetic tree generated by IQtree of TIR domains from Clade 8. Clade 8 was a mainly bacterial clade into which a subset of Metazoan TIRs, notably SARM1, clustered. **C.** A circular maximum likelihood phylogenetic tree generated by IQtree of TIR domains from Clade 4. This clade contained all of the TIR domains from land plants (Embryophyta). White circles indicate notable TIR domain-containing proteins: (**AB**) *Hs = Homo sapiens,* (**C**) *At = Arabidopsis thaliana*, *Al = Arabidopsis lyrata, Bd =Brachypodium distachyon, Nb = Nicotiana bethamiana.* Both leaf color and strip color indicate the eukaryotic supergroup. Wedges represent collapsed parts of the phylogenetic trees. Trees are rooted on outgroup sequences (gray wedges). Ultrafast bootstrap values calculated by IQtree at all nodes with support >70 are shown as black dots.

bioRxiv preprint doi: https://doi.org/10.64898/2026.04.30.722052; this version posted May 22, 2026. The copyright holder for this preprint (which was not certified by peer review) is the author/funder, who has granted bioRxiv a license to display the preprint in perpetuity. It is made available under a CC-BY 4.0 International license.

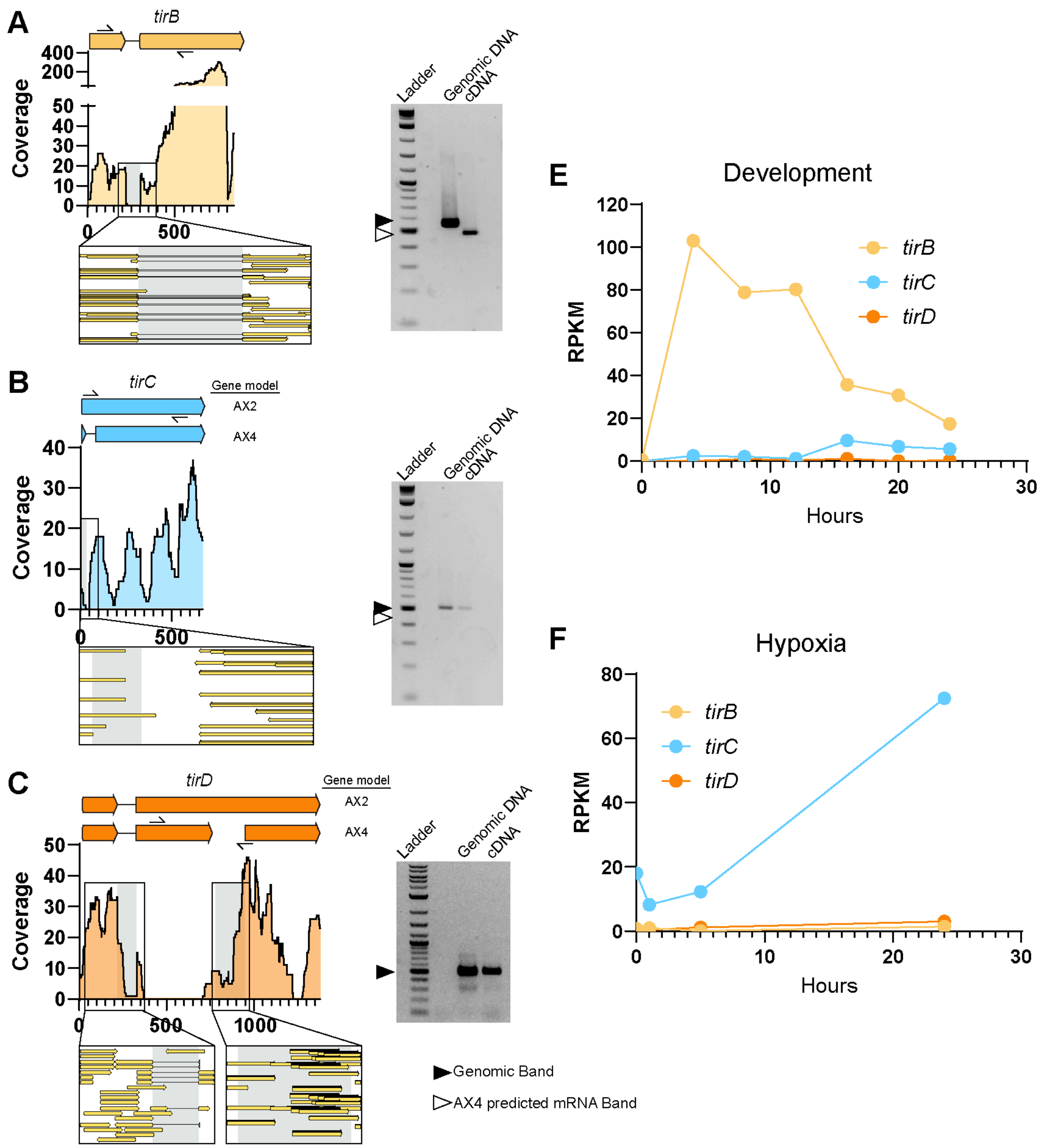

Figure S4. *tirBCD* gene models

A-C. Diagrams of predicted gene models for *tirB* (**A**), *tirC* (**B**), and *tirD* (**C**). For each gene, the AX2 and AX4 annotations are shown to scale. For *TirB* the two annotations agree and so a single schematic of the coding sequence is shown. For *TirD*, the AX4 annotation predicted that the gene was two genes (*DDB_G028737* and *DDB_G0287321*). Below each schematic is a plot showing the coverage via RNAseq at each position on the genomic locus. The focus box zooms in on the indicated area to show the alignment of individual reads to the genomic locus. The gray boxes indicate the predicted introns by both AX2 and AX4 and are displayed on both the coverage plots and the focus boxes. To the right of the RNAseq cover plots is an Agarose gel picture where the PCR products of the primers indicated on the gene locus schematics were used to amplify gDNA or cDNA. The black arrow indicates the expected product size when

bioRxiv preprint doi: https://doi.org/10.64898/2026.04.30.722052; this version posted May 22, 2026. The copyright holder for this preprint (which was not certified by peer review) is the author/funder, who has granted bioRxiv a license to display the preprint in perpetuity. It is made available under a CC-BY 4.0 International license.

amplifying genomic DNA while the empty arrow shows the product size of amplified cDNA based on the AX4 genome annotation. For *TirD*, the reverse primer spans the AX4-predicted intron-exon boundary and so no product is predicted. **E.** Reads per Kilobase of transcript per Million reads mapped (RPKM) of *TirB, TirC,* and *TirD* (*DDB_G0287321*) from Parikh et. al. [46] over 24 hours of development on a filter. **F.** Reads per Kilobase of transcript per Million reads mapped (RPKM) of *TirB, TirC,* and *TirD* (*DDB_G0287321*) from Hesnard et. al. [45] over 24 hours of hypoxia exposure.

bioRxiv preprint doi: https://doi.org/10.64898/2026.04.30.722052; this version posted May 22, 2026. The copyright holder for this preprint (which was not certified by peer review) is the author/funder, who has granted bioRxiv a license to display the preprint in perpetuity. It is made available under a CC-BY 4.0 International license.

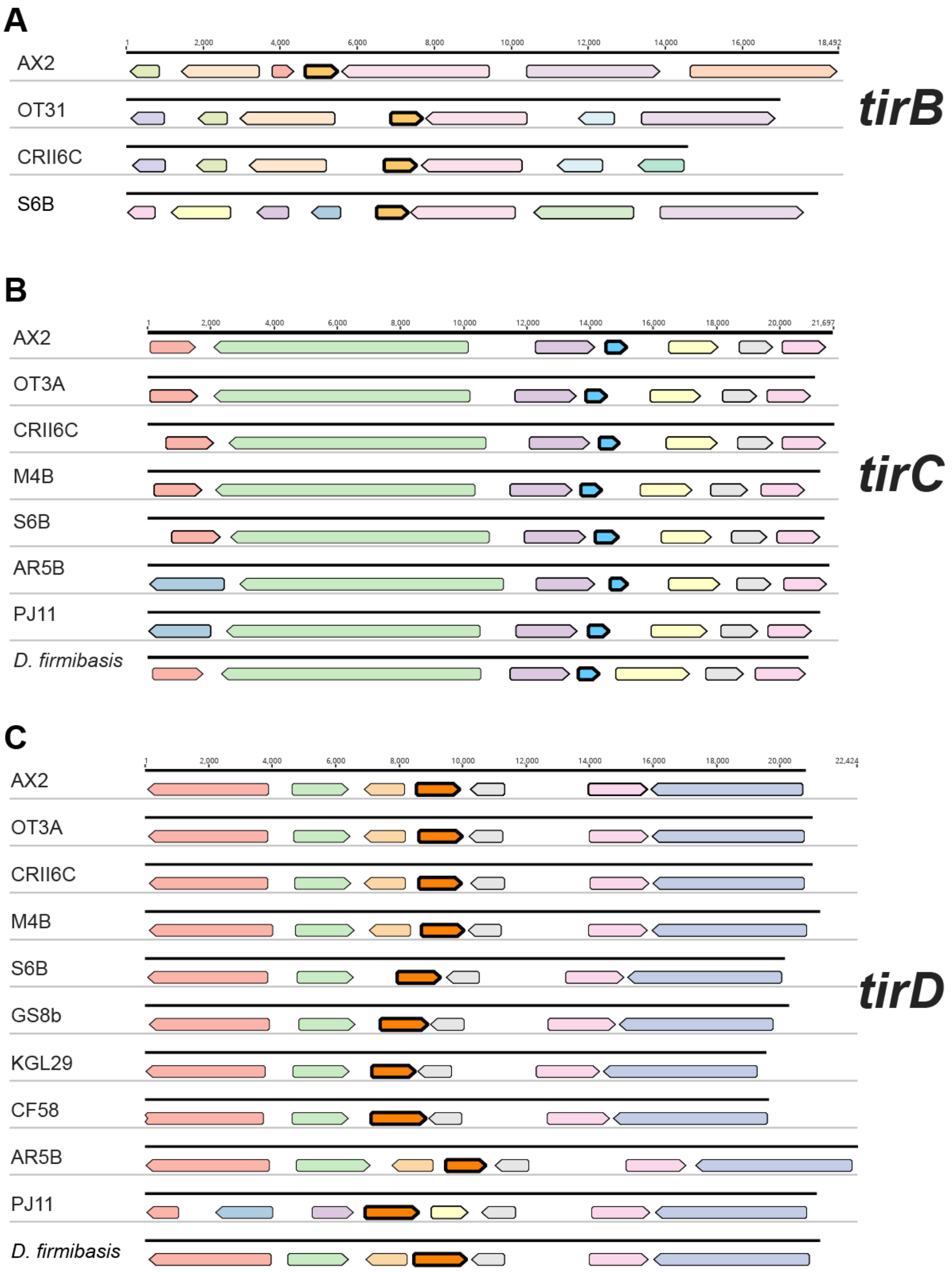

Figure S5. Syntenic distribution of genes at TirB, TirC, TirD genomic loci
Genetic synteny across Dicyostelium species at the (**A**) TirB, (**B**) TirC, and (**C**) TirD loci. Colored arrows represent homologous ORFs across the genomes, with strain identifiers listed on the left side. Genes of the same color within a given locus are homologous. Black outlined arrow represents the TIR-domain containing gene. Numbers above the loci show the nucleotide scale of the locus and genes.

bioRxiv preprint doi: https://doi.org/10.64898/2026.04.30.722052; this version posted May 22, 2026. The copyright holder for this preprint (which was not certified by peer review) is the author/funder, who has granted bioRxiv a license to display the preprint in perpetuity. It is made available under a CC-BY 4.0 International license.

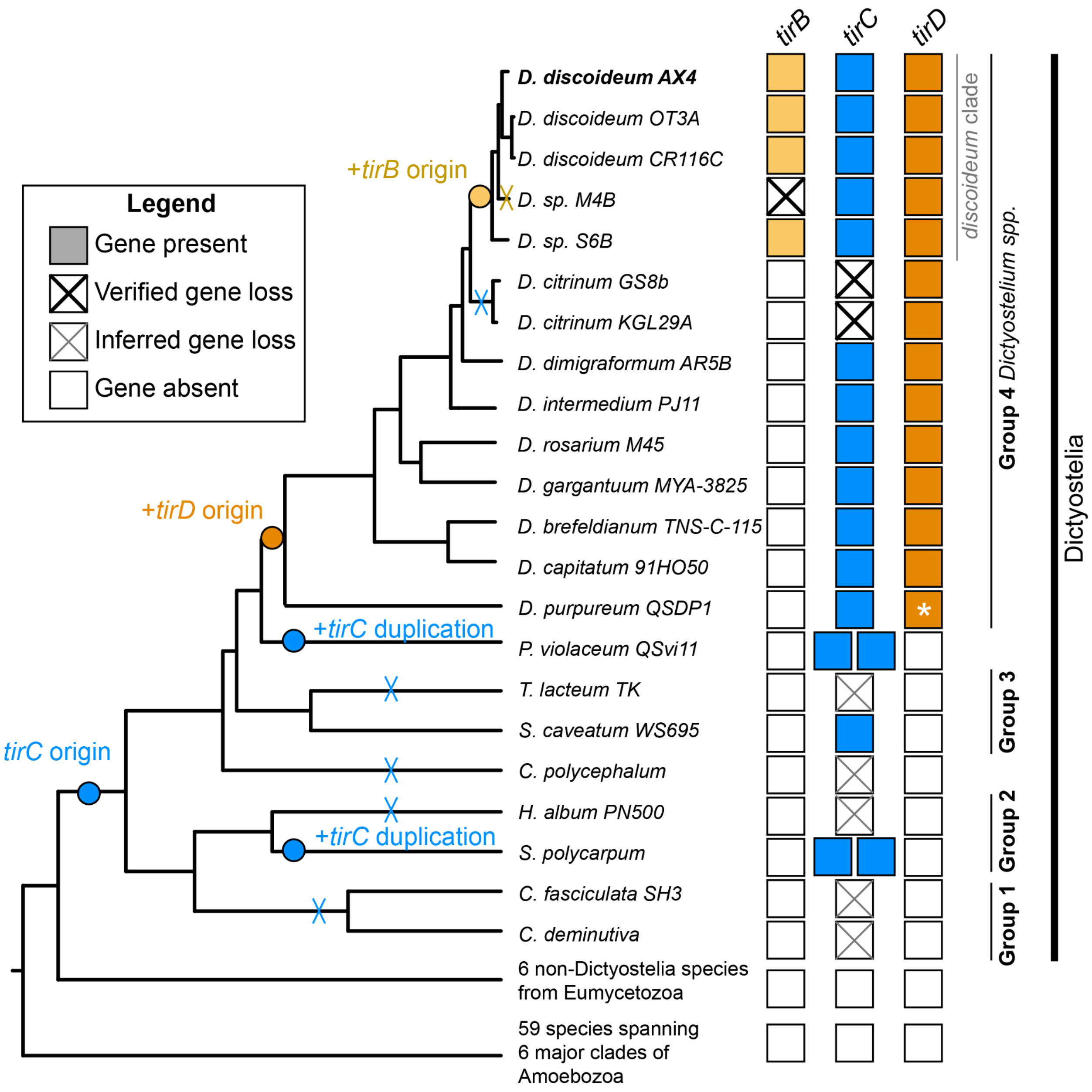

Figure S6. Origin and duplications of *tirBCD* genes within Dictyostelia
Colored boxes indicate species of cellular slime mold amoebae where *tirB*, *tirC*, and *tirD* genes were present, based on tBLASTn and TIR HMM searches of the amoeba genomes. All colored boxes were discovered by tBLASTn or both methods, except for *D. purpureum* TirD (*), which was identified only by a TIR HMM search. Black Xs mark instances of gene loss where we identified either pseudogenes or deletions in the syntenic locus. Gray Xs mark inferred losses based on the phylogeny, although these apparent absences could result from incomplete assemblies. Outgroup Amoebozoa and Eumycetozoa datasets searched included a combination of genomes and transcriptomes within Eukprot v3. Species tree is based on Schilde et. al. [47], branch lengths not to scale.

bioRxiv preprint doi: https://doi.org/10.64898/2026.04.30.722052; this version posted May 22, 2026. The copyright holder for this preprint (which was not certified by peer review) is the author/funder, who has granted bioRxiv a license to display the preprint in perpetuity. It is made available under a CC-BY 4.0 International license.

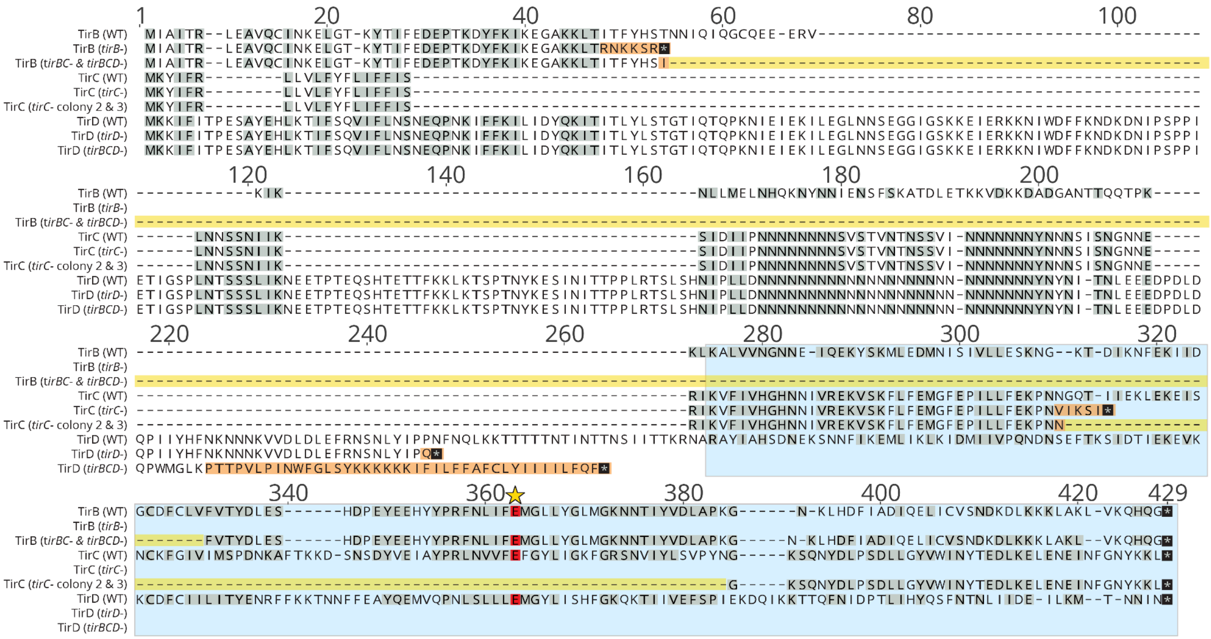

Figure S7. Amino acid alignment of TirB, TirC, and TirD and their mutants

Amino acid alignment of wild type TirB, TirC, and TirD and the predicted protein products made by genetically modified strains. For each protein, the wild type sequence is listed first, followed by predicted protein products of the mutant clones. The blue box indicates the TIR domain of the proteins. The orange boxes indicate frameshift induced mutations in the protein. The yellow box highlights the amino acids of the protein that have been deleted. Stop codons are highlighted with a black box. The catalytic glutamate is designated with a red box and the locus is marked with a yellow star.

bioRxiv preprint doi: https://doi.org/10.64898/2026.04.30.722052; this version posted May 22, 2026. The copyright holder for this preprint (which was not certified by peer review) is the author/funder, who has granted bioRxiv a license to display the preprint in perpetuity. It is made available under a CC-BY 4.0 International license.

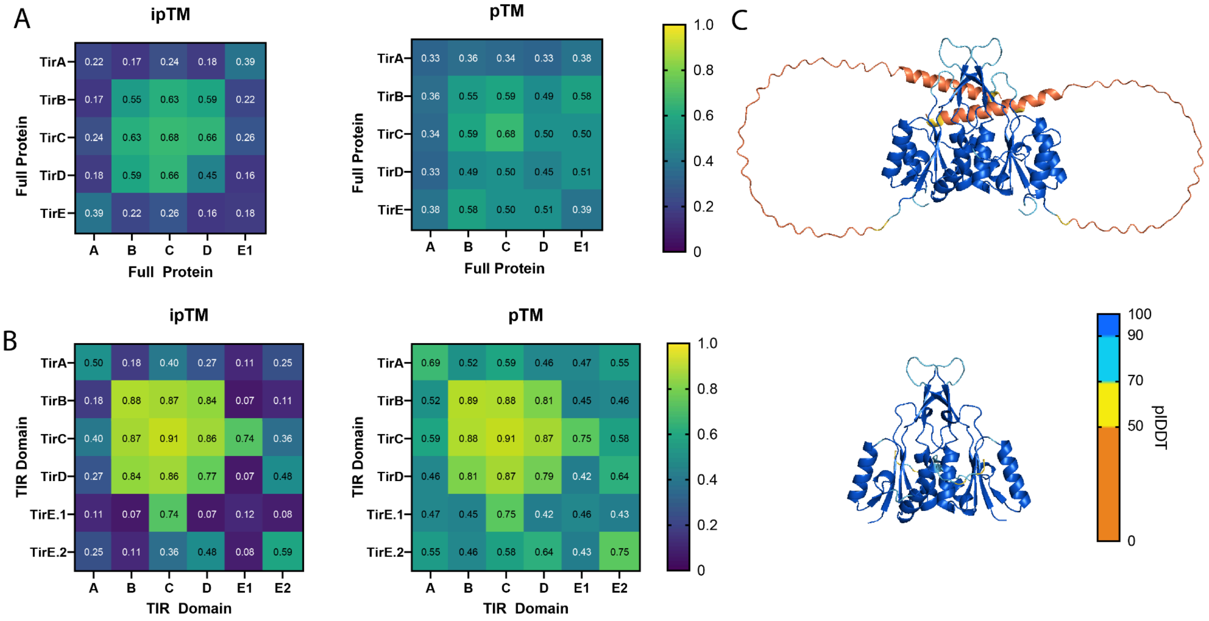

Figure S8. TirC is predicted to homodimerize

A. ipTM and pTM scores of AlphaFold Multimer predictions of *D. discoideum* TIR full protein dimers. **B.** ipTM and pTM scores of AlphaFold Multimer predictions of *D. discoideum* TIR domain dimers.**C.** AlphaFold models of full length (top) and TIR domain (bottom) TirC homodimers. Residues are colored by pIDDT score.

bioRxiv preprint doi: https://doi.org/10.64898/2026.04.30.722052; this version posted May 22, 2026. The copyright holder for this preprint (which was not certified by peer review) is the author/funder, who has granted bioRxiv a license to display the preprint in perpetuity. It is made available under a CC-BY 4.0 International license.

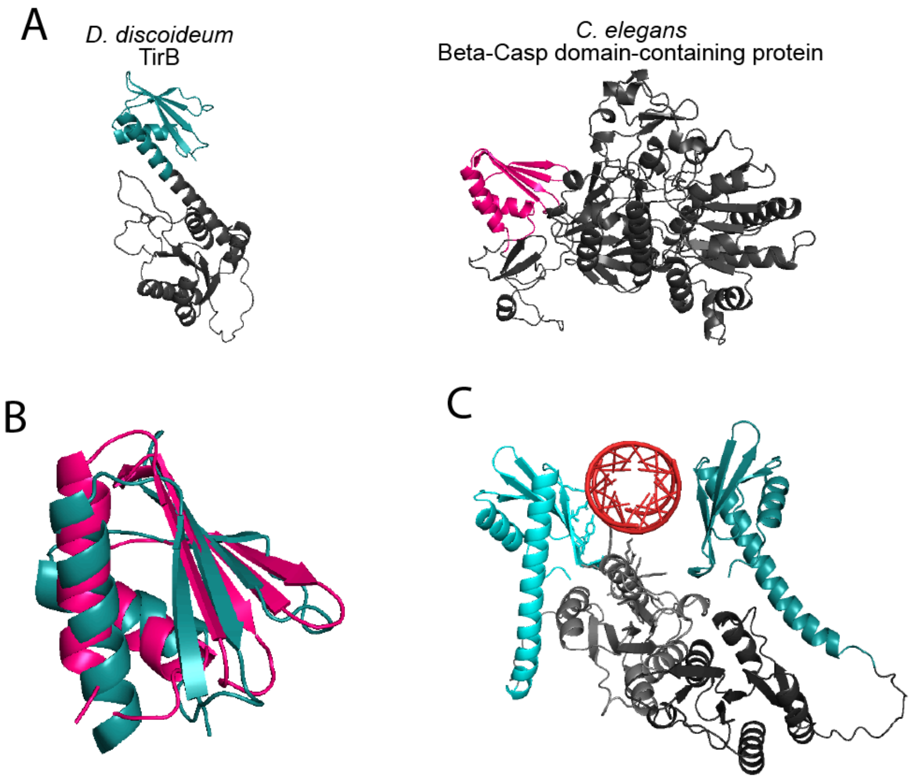

Figure S9. Predicted RNA binding in TirB's N-terminus

A. AlphaFold predictions of *D. discoideum* TirB and *C. elegans* Beta-Casp domain-containing protein, the highest scoring FoldSeek hit for TirB's N-terminus. The similar domains are colored, teal in TirB, and pink partial KH domain. **B.** Structural alignment of the KH domain of *C. elegans* Beta-Casp domain-containing protein and TirB's N-terminus. The two structures aligned with an RMSD of 3.8 over 45 atoms. **C.** AlphaFold models of full length TirB-TirB homodimers binding a dsRNA molecule. dsRNA is shown in red, while the N-Termini of TirB are shown in shades of teal, and the TIR domain in gray. TirBs are differentiated by shading (protomer 1 is light, protomer 2 is dark).

bioRxiv preprint doi: https://doi.org/10.64898/2026.04.30.722052; this version posted May 22, 2026. The copyright holder for this preprint (which was not certified by peer review) is the author/funder, who has granted bioRxiv a license to display the preprint in perpetuity. It is made available under a CC-BY 4.0 International license.

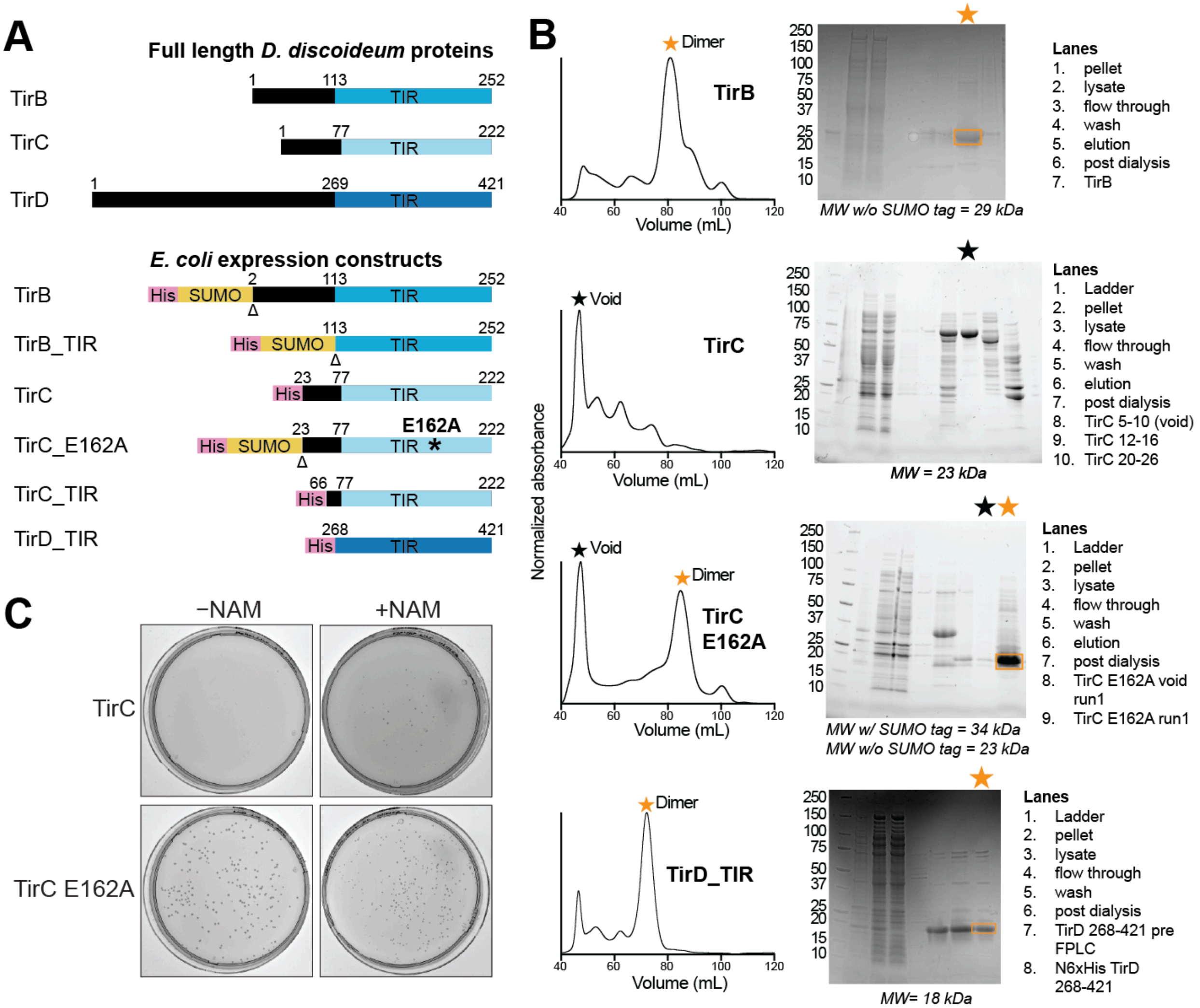

Figure S10. Purification of TirB, TirC, and TirD

A. Schematic of full length proteins and constructs. The SUMO2 tag was proteolytically removed before experimentation. His-tagged proteins had GS linkers between the tag and the protein. Numbers indicate the amino acid coordinates of TirB, C, and D proteins that were included. The TirC construct excluded the N-terminal hydrophobic alpha helix to facilitate protein expression and purification. * shows the location of the E162A point mutation. Δ indicates the SUMO2 cleavage site. **B.** Recombinant protein was purified via size exclusion chromatography (traces on left) and purity of fractions were determined by SDS-PAGE (gel images on right). Lanes are numbered from left to right. Stars indicate primary fractions considered for activity assays. Note that for WT TirC there was no obvious band on the gel and yet the void fraction contains measurable NADase activity (as shown in other figures). Contaminants near 20 kDa and 75 kDa are common E. coli proteins which bind nickel-resin with appreciable affinity under the conditions tested. **C.** TirC is toxic to E. coli, but partially rescued by extracellular nicotinamide. Images show colonies of bacteria on MDG agar plates in the absence of inducer, with toxicity due to leaky TirC expression from the T7 promoter. The E162A TirC mutant construct was not toxic.

bioRxiv preprint doi: https://doi.org/10.64898/2026.04.30.722052; this version posted May 22, 2026. The copyright holder for this preprint (which was not certified by peer review) is the author/funder, who has granted bioRxiv a license to display the preprint in perpetuity. It is made available under a CC-BY 4.0 International license.

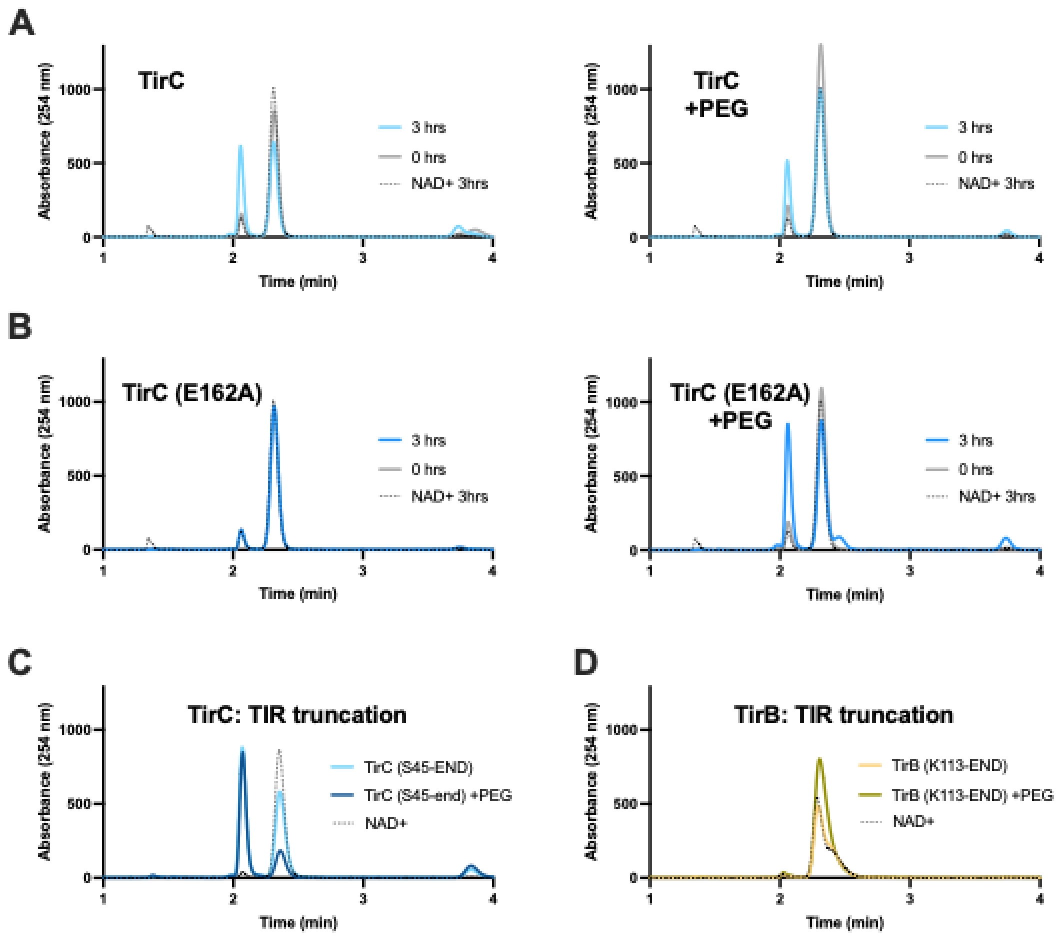

Figure S11. NAD$^+$ processing by TirC and TirB mutants in the presence of PEG 8000
A. Time course of *in vitro* co-incubations of NAD$^+$ and WT TirC in the presence of PEG 8000 as a molecular crowding agent. In both cases, WT TirC cleaves NAD$^+$ to produce ADPR and NAM. Black dotted lines show the absorbance of NAD$^+$ alone, following 3h incubation in the study conditions. Colored lines show traces after 0-3h co-incubation with protein. **B.** Similar time course using the TirC E162A mutant, which cleaves NAD$^+$ only in the presence of PEG 8000. **C.** TirC truncation mutant (S45-END) that includes only the TIR domain processes NAD$^+$ similar to TirC WT, with only a slight change in processing rate in the presence of PEG. **D.** Truncation mutant of TirB (K113-END) to the TIR domain does not show any NAD$^+$ cleavage activity, even in the presence of PEG. Similar results were obtained for the TIR domain of TirD (shown in Fig 3).

bioRxiv preprint doi: https://doi.org/10.64898/2026.04.30.722052; this version posted May 22, 2026. The copyright holder for this preprint (which was not certified by peer review) is the author/funder, who has granted bioRxiv a license to display the preprint in perpetuity. It is made available under a CC-BY 4.0 International license.

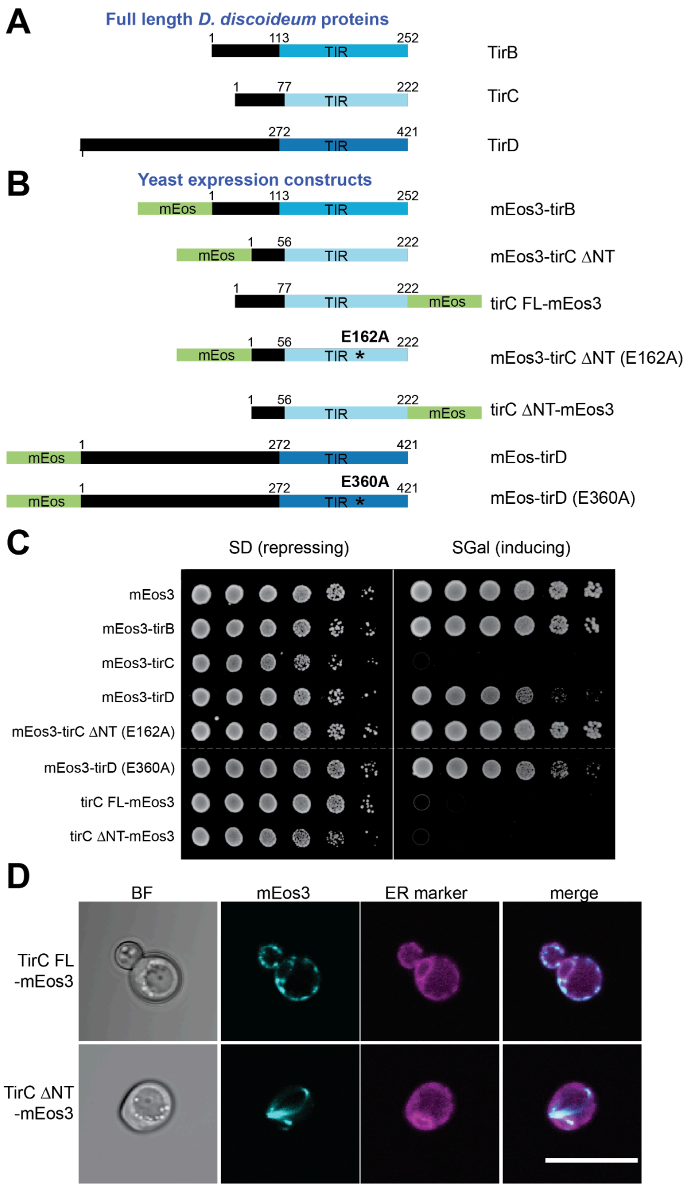

Figure S12. Expression of TirBCD in yeast

A & B. Schematic of *D. discoideum* TIR proteins expressed in *S. cerevisiae*. Numbers indicate the coordinates of TirB, C, and D proteins that were included. FL = full-length, ΔNT = N-terminal helix is missing, * shows the location of the putative catalytic glutamate. To facilitate DAmFRET and protein localization studies, the amoeba proteins were tagged with mEos fluorophores. **C**. Viability assay of yeast carrying the TirBCD expression constructs were serially diluted (5-fold) and spotted onto selective media either to repress (SD) or induce (Sgal) protein expression. **D.** Localization of TirC FL or ΔNT proteins in yeast expressing the mTagBFP2 ER marker. Cells were grown in excess nicotinamide to enhance TirC expression. TirC FL strongly co-localizes with the ER but TirC ΔNT does not, suggesting that the N-terminal helix facilitates membrane localization.

bioRxiv preprint doi: https://doi.org/10.64898/2026.04.30.722052; this version posted May 22, 2026. The copyright holder for this preprint (which was not certified by peer review) is the author/funder, who has granted bioRxiv a license to display the preprint in perpetuity. It is made available under a CC-BY 4.0 International license.

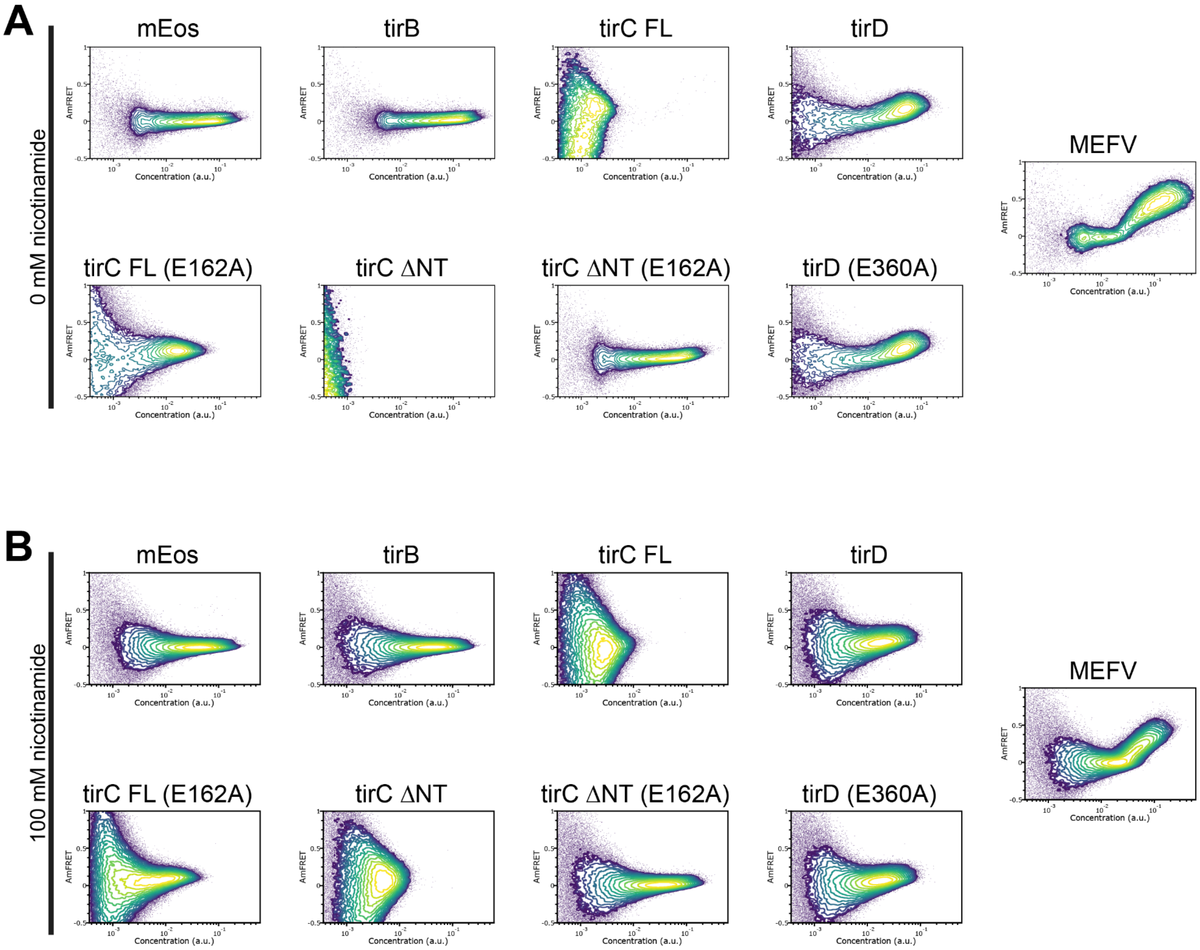

Figure S13. Self oligomerization of TIR proteins via DAmFRET

Representative DAmFRET data showing the propensity of various *D. discoideum* TIR constructs to oligomerize in yeast cells. mEos serves as a negative control, which fails to aggregate even at high concentrations, while MEFV is a positive control for aggregation and filamentation. Cells were grown without supplemental nicotinamide (**A**) and with 100 mM nicotinamide (**B**).

bioRxiv preprint doi: https://doi.org/10.64898/2026.04.30.722052; this version posted May 22, 2026. The copyright holder for this preprint (which was not certified by peer review) is the author/funder, who has granted bioRxiv a license to display the preprint in perpetuity. It is made available under a CC-BY 4.0 International license.

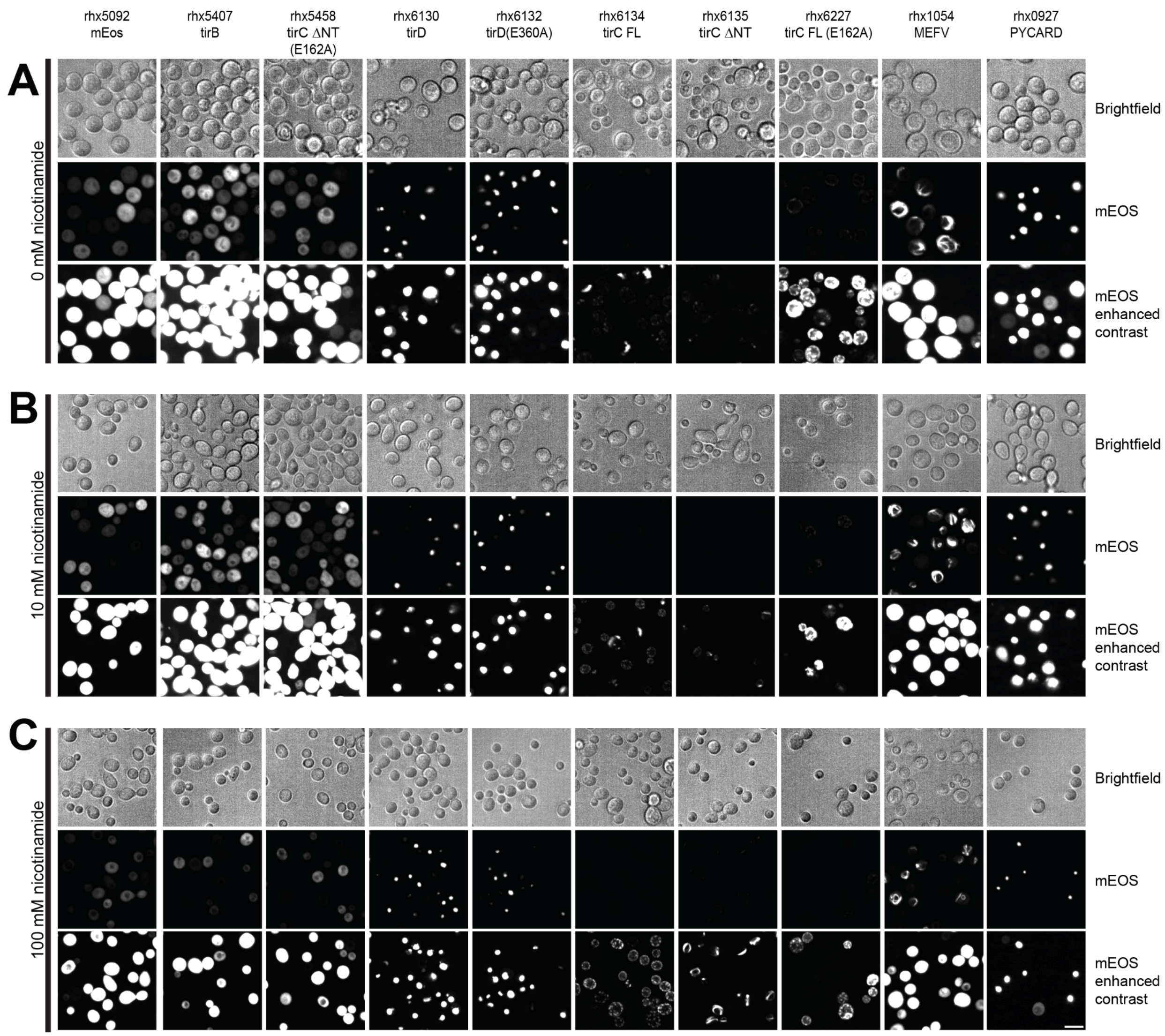

Figure S14. TIR construct localization in yeast

Microcopy images of yeast cells expressing various mEos3-tagged constructs. Images are from brightfield and the green (mEos3) channel. The mEOS channel is displayed twice with the contrast on the second image being enhanced to allow visualization of low mEos3 expression. Yeast cells were grown without supplemental nicotinamide (**A**), with 10 mM nicotinamide (**B**), and with 100 mM nicotinamide (**C**). Scale bar is 10 µm.

bioRxiv preprint doi: https://doi.org/10.64898/2026.04.30.722052; this version posted May 22, 2026. The copyright holder for this preprint (which was not certified by peer review) is the author/funder, who has granted bioRxiv a license to display the preprint in perpetuity. It is made available under a CC-BY 4.0 International license.

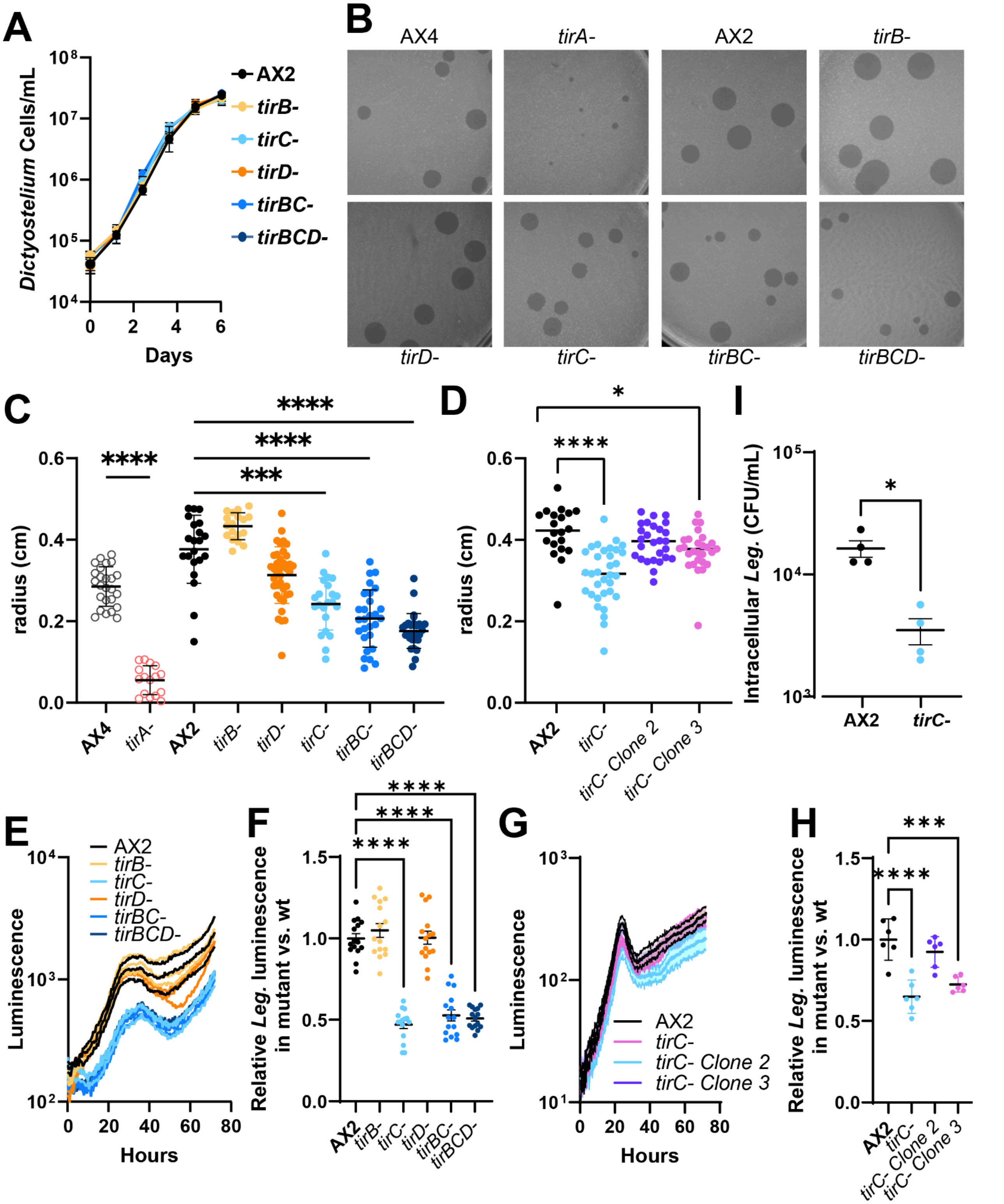

1287 **Figure S15. *tirC-* mutants are defective in bacterial uptake**

1288 **A.** Growth curve of wild type (AX2) and TIR mutant *D. discoideum* cultures in HL5 rich media.
1289 The TirBCD proteins are not essential for cell viability or growth. **B.** Representative photos of *D.*

bioRxiv preprint doi: https://doi.org/10.64898/2026.04.30.722052; this version posted May 22, 2026. The copyright holder for this preprint (which was not certified by peer review) is the author/funder, who has granted bioRxiv a license to display the preprint in perpetuity. It is made available under a CC-BY 4.0 International license.

discoideum plaque sizes on a lawn of *K. pneumoniae* measured after four days of growth for all *D. discoideum* strains. **C-D.** Quantification of plaques across three replicate plates per genotype, measured after four days. Panel **C** shows plaque size across multiple genotypes while panel **D** shows plaque size variation amongst different *tirC*- clones. **E,G.** Curves of luminescent *Legionella pneumophila* (Leg) during infection of *D. discoideum* TIR mutants over 72 hours. *D. discoideum* cells were infected with Leg at an MOI of 10 and incubated at 22°C for three days. Experiment was carried out in triplicate wells with the luminescence curve of each well plotted. **F,H.** Comparison of the peak luminescence relative to wild-type reached by each strain between the 20-40 hours post infection window. Panel **E-F** shows Leg luminescence across multiple genotypes while panel **G-H** shows variation amongst different *tirC*- clones. **I.** Gentamicin protection assay during Leg infection of AX2 and *tirC*-. Error bars show standard deviations. Statistical significance for C,D, was determined by one-way ANOVA with Kruskal-Wallis posttest and statistical support for F,H was determined by one-way ANOVA with Kruskal-Wallis posttest; $*p \le .05$,$***p \le .001$, $****p \le .0001$. Statistical significance for I was determined by two tailed student's t-test; $*p \le .05$.

bioRxiv preprint doi: https://doi.org/10.64898/2026.04.30.722052; this version posted May 22, 2026. The copyright holder for this preprint (which was not certified by peer review) is the author/funder, who has granted bioRxiv a license to display the preprint in perpetuity. It is made available under a CC-BY 4.0 International license.

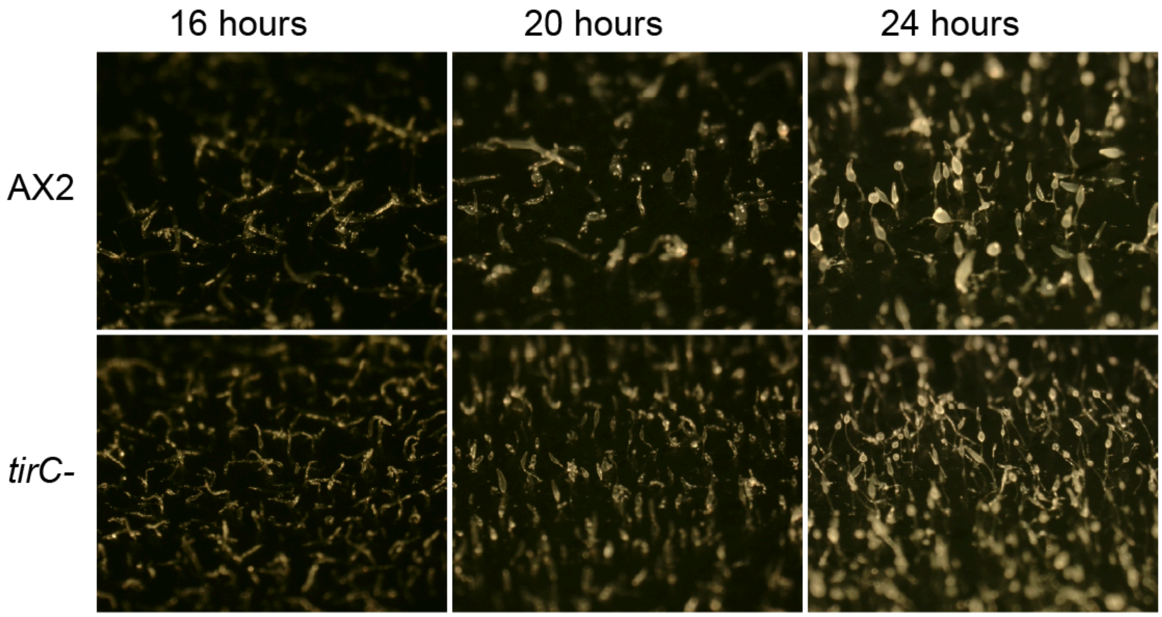

Figure S16. Development of fruiting bodies

Representative photographs of the development of fruiting bodies in *D. discoideum* wild type (AX2) and *tirC-* over 24 hours on SM/5 agar plates with 0.5% charcoal. *D. discoideum* cells aggregate into distinct morphological forms during the transition from unicellular to multicellular states. At 16 hours for both AX2 and *tirC-,* a mixture of slugs (horizontal rods) and fingers (vertical rods) are observed. At 20 hours, early culminants are visible, which are characterized by an asymmetrical main body that is smaller at the apical tip, held aloft by a thin stalk. At 24 hours, a mixture of mature fruiting bodies, with spherical sori atop thin stalks, and early culminants are visible for both strains.

bioRxiv preprint doi: https://doi.org/10.64898/2026.04.30.722052; this version posted May 22, 2026. The copyright holder for this preprint (which was not certified by peer review) is the author/funder, who has granted bioRxiv a license to display the preprint in perpetuity. It is made available under a CC-BY 4.0 International license.

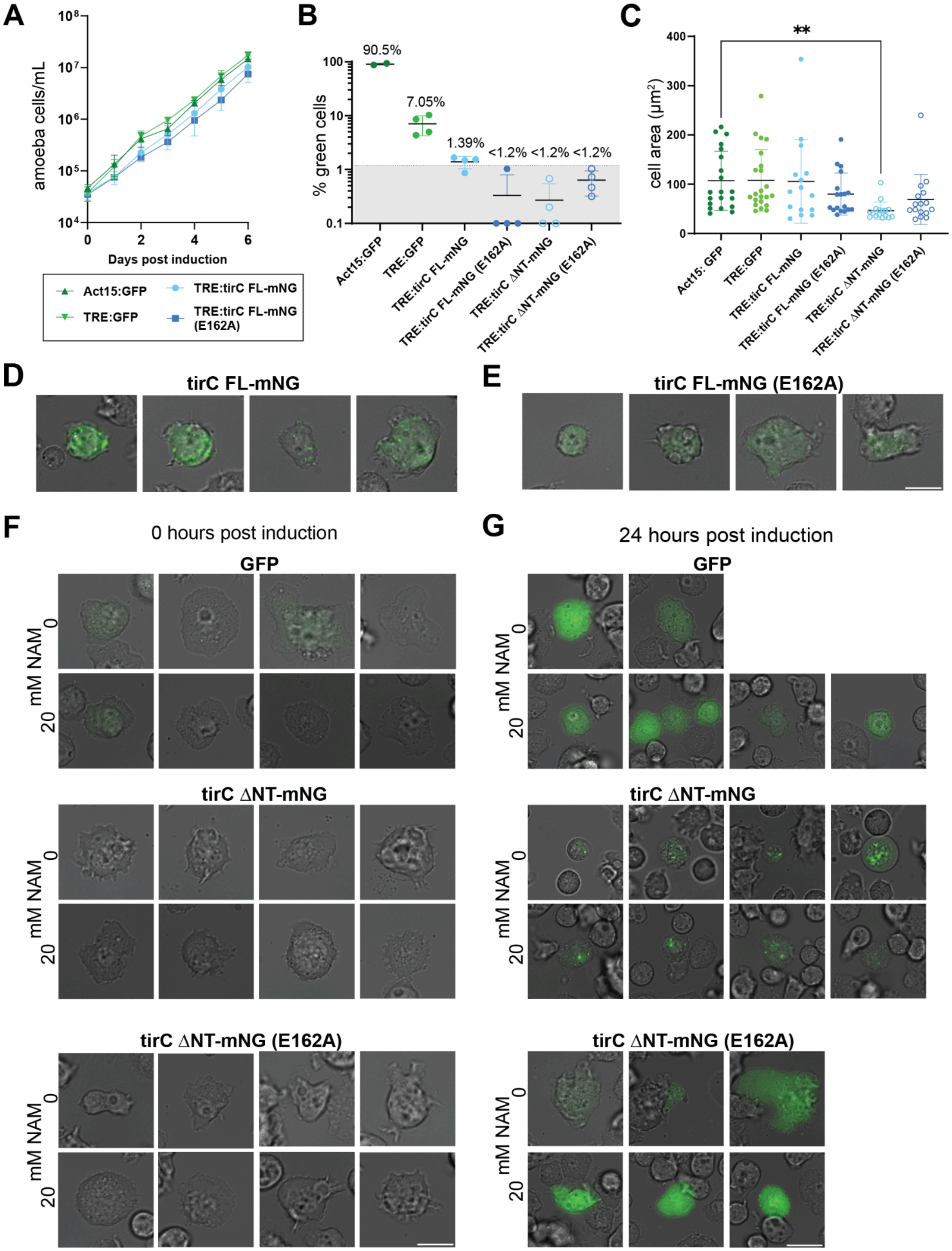

1316

1317 **Figure S17. TirC overexpression in *D. discoideum***

1318 **A.** Cell growth assay for amoebae expressing proteins under constitutive (Act15) or inducible
1319 (TRE) promoters. Doxycycline was removed from the cells at Day 0 to induce overexpression of
1320 polyclonal transformants expressing mNeonGreen-tagged (mNG) TirC or GFP controls. All
1321 overexpression cell lines grew at a similar rate. **B.** Quantification of green fluorescent cells in the

bioRxiv preprint doi: https://doi.org/10.64898/2026.04.30.722052; this version posted May 22, 2026. The copyright holder for this preprint (which was not certified by peer review) is the author/funder, who has granted bioRxiv a license to display the preprint in perpetuity. It is made available under a CC-BY 4.0 International license.

polyclonal populations. Each dot represents at least 80 counted cells. Shaded area shows the approximate limit of detection, with symbols on the x-axis indicating fields of view that had no detectable green cells. **C.** Quantification of cross-sectional cell area for overexpression cell lines. Statistical significance was tested by a one-way ANOVA with Kruskal-Wallis posttest with Dunn's multiple corrections, which indicated that only the TirC ΔNT cells were significantly smaller than the Act15:GFP controls. ** = $p \leq 0.001$ **D.** Microscopy of amoeba cells expressing full-length (FL) WT TirC and **E.** FL E162A TirC. WT TirC overexpression cell lines exhibited puncta while E162A TirC overexpression cells show more diffuse distribution of fluorescent signal. Images were taken 6 days post-induction. **F.** Microscopy of amoeba cells expressing inducible GFP or inducible TirC ΔNT (both WT and E162A) at 0 hours after induction and Nicotinamide (NAM) supplementation. At 0 hours post induction, the inducible GFP control cells show little to no green fluorescence indicating minimal leaky expression. No green fluorescence was observed in the TirC ΔNT overexpression cell lines. Cells exhibited amoeboid morphology and substrate adhesion characteristic of healthy cells. **G.** Microscopy of amoeba cells expressing N-terminal truncations (ΔNT) of TirC at 24 hours after induction and NAM supplementation. At 24 hours post induction, the inducible GFP control cells show diffuse green fluorescence and normal cell morphology. In contrast, cells expressing TirC ΔNT are rounded and detached (morphology and behavior associated with loss of viability and cell death) when cultured without NAM. However, this same culture grew as amoeboid, adhered cells observed in the 20mM NAM supplemented conditions. Cells expressing ΔNT E162A TirC exhibited normal morphology, adhesion, and diffuse distribution of green fluorescent signal regardless of NAM concentration in media. Exposure and contrast levels were matched across samples. Scale bar is 10 µm.

bioRxiv preprint doi: https://doi.org/10.64898/2026.04.30.722052; this version posted May 22, 2026. The copyright holder for this preprint (which was not certified by peer review) is the author/funder, who has granted bioRxiv a license to display the preprint in perpetuity. It is made available under a CC-BY 4.0 International license.

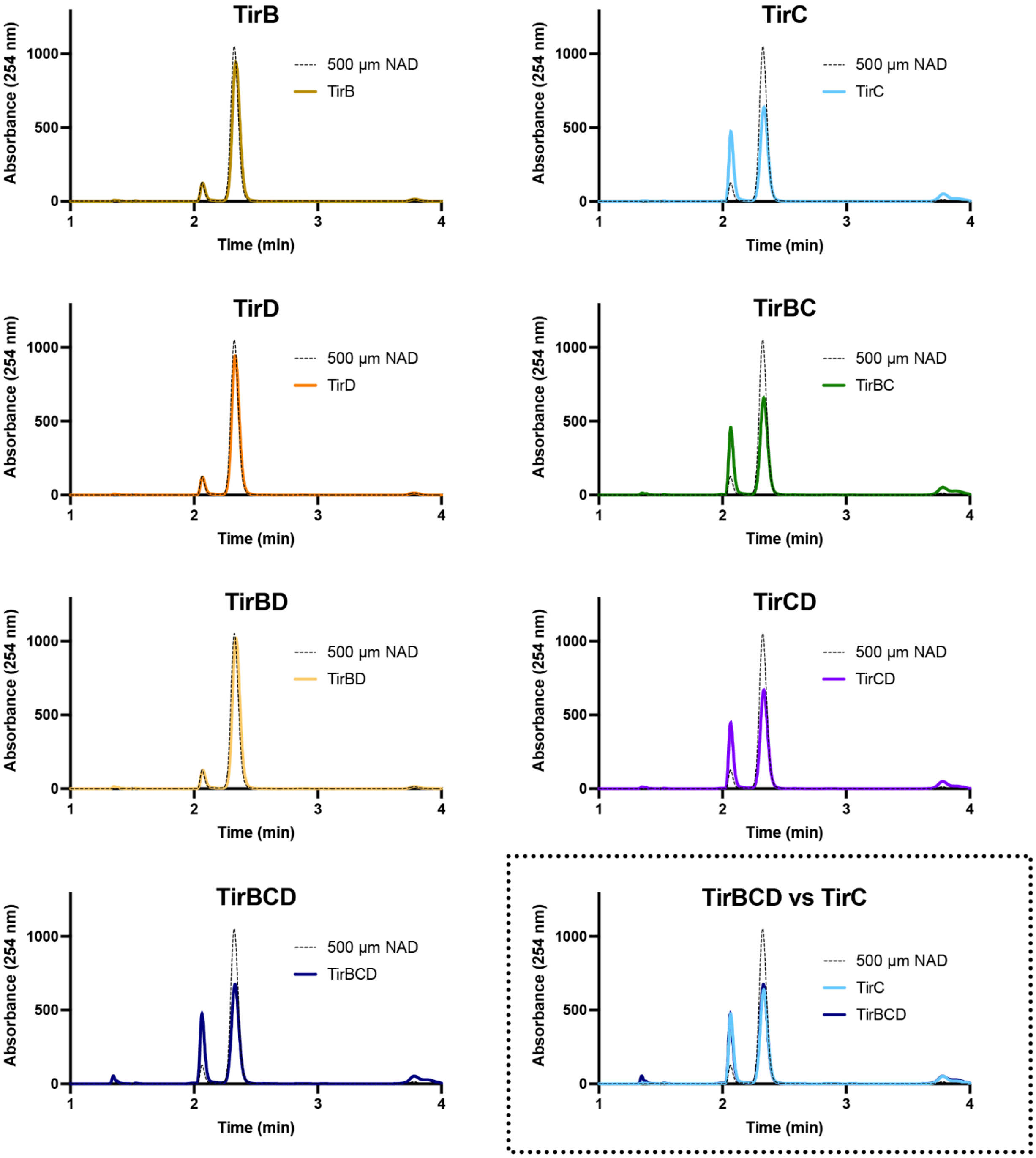

Figure S18. Co-incubation of TirB, C, and D proteins *in vitro* does not alter NADase activity

High-performance liquid chromatography analysis of NAD^+ incubated with purified TirB, TirC, TirD, or combinations thereof. We observed no NAD^+ processing from TirB or TirD, alone or with each other. In all other mixes, the observed activity was equivalent to the activity of TirC alone. The graph in the dotted box overlays the TirC-only results with those from the TirB, C, and D mix, showing that they are nearly identical.

bioRxiv preprint doi: https://doi.org/10.64898/2026.04.30.722052; this version posted May 22, 2026. The copyright holder for this preprint (which was not certified by peer review) is the author/funder, who has granted bioRxiv a license to display the preprint in perpetuity. It is made available under a CC-BY 4.0 International license.

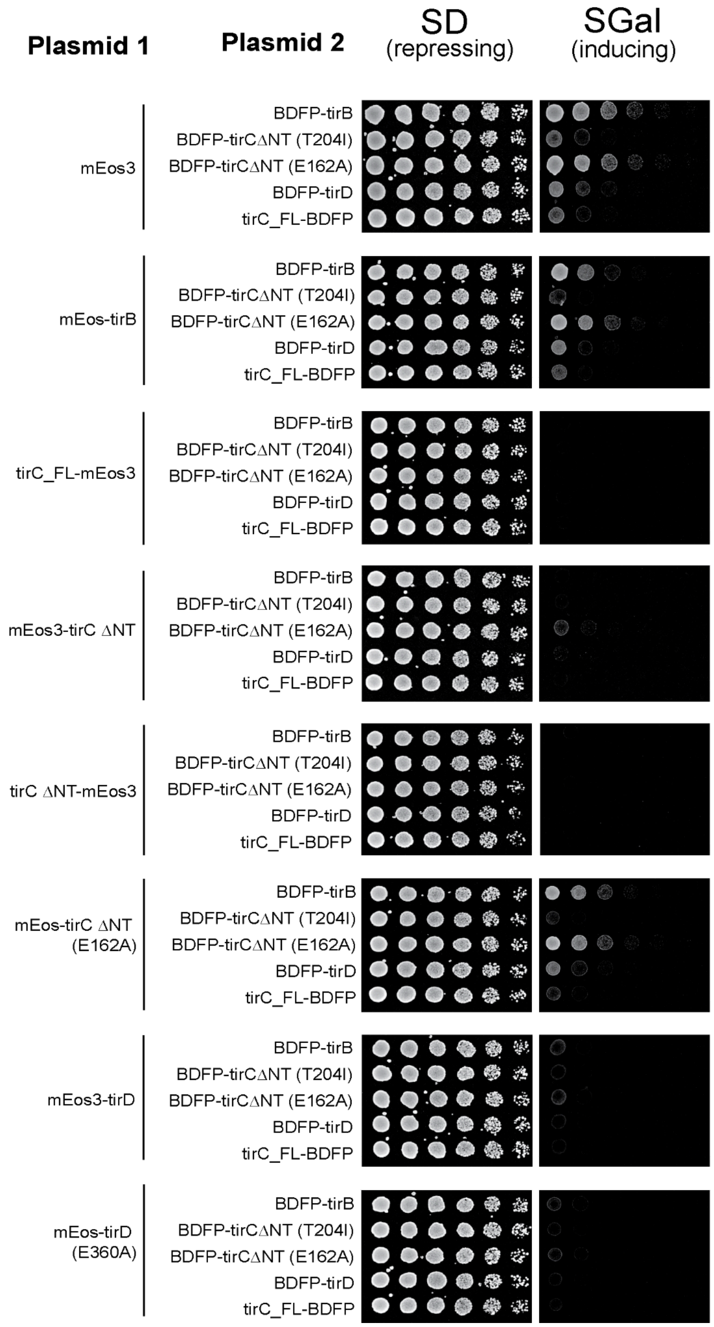

Figure S19. Toxicity of TirBCD co-expression in *S. cerevisiae*
Yeast cells carrying the indicated plasmid constructs were serially diluted (5-fold) and spotted onto selective media to either repress or induce co-expression of the indicated mEos- and BDFP-tagged proteins. TirC constructs were either full length (FL) or truncated to remove the N-terminal helix (ΔNT). Plates were incubated at 30ºC for 3 days. Images are representative of 2 replicates of the spotting assay.
