# Supplementary material for: Eukaryotic domestication of a bacterial immune protein following horizontal transfer
Source: bioRxiv. 2026 May 5:2026.04.30.722052. Preprint. [Version 2] doi: 10.64898/2026.04.30.722052 (PMC13142454; doi:10.64898/2026.04.30.722052)
Supplement: Supplementary file 1 [file NIHPP2026.04.30.722052v2-supplement-7.pdf]

bioRxiv preprint doi: https://doi.org/10.64898/2026.04.30.722052; this version posted May 5, 2026. The copyright holder for this preprint (which was not certified by peer review) is the author/funder, who has granted bioRxiv a license to display the preprint in perpetuity. It is made available under aCC-BY 4.0 International license.

Supplemental Figures, Tables, and Movie

Legend: Supp. Table. 1: **TIR domain containing proteins from phylogenetic analysis.**
A table displaying information on all proteins in the phylogenetic analysis (Fig. 1). Data includes the sequence name, full protein amino acid sequence, the domains present, the clade in the phylogenetic tree of Fig. 1, taxonomic information, and which domain of life the sequence is from. Domain information is displayed as <Name of the sequence>, <total length of protein>, <start of domain> | <end of domain> | <domain name> | <domain Pfam ID>. Multiple domains are separated by a comma.

Legend: Supp. Table. 2: **Neighboring genes in the *tirB, tirC,* and *tirD* loci.**
The neighboring genes (3 upstream, 3 downstream) around *tirB, tirC,* and *tirD* in the *Dictyostelium discoideum* AX2 genome are identified by the TIR locus, the name of the gene, the position of the gene relative to the nearby TIR gene, the gene annotation, and gene symbol. We searched both the genomic nucleotide sequences as well as the exonic sequences with nBLAST and display the top hit result in the table. Because we excluded all Dictyostelia, many of these genes yielded no BLAST hits (labeled as “No hit found”). We next translated all of these nucleotide sequences into amino acid sequences and used Hmmscan to determine what domains are present and used Jackhmmer to look for homology to other proteins. From Jackhmmer, we are reporting the taxonomic classification (at the domain level) of the significant hits. We then show the nucleotide sequences (both the genomic sequence and the exon-only sequence). Finally, we display the protein sequence.

Legend: Supp. Table. 3: **Strains used in Study**

Legend: Supp. Table. 4: **Plasmids used in Study**

Legend: Supp. Table. 5: **Primers used in Study**

bioRxiv preprint doi: https://doi.org/10.64898/2026.04.30.722052; this version posted May 5, 2026. The copyright holder for this preprint (which was not certified by peer review) is the author/funder, who has granted bioRxiv a license to display the preprint in perpetuity. It is made available under aCC-BY 4.0 International license.

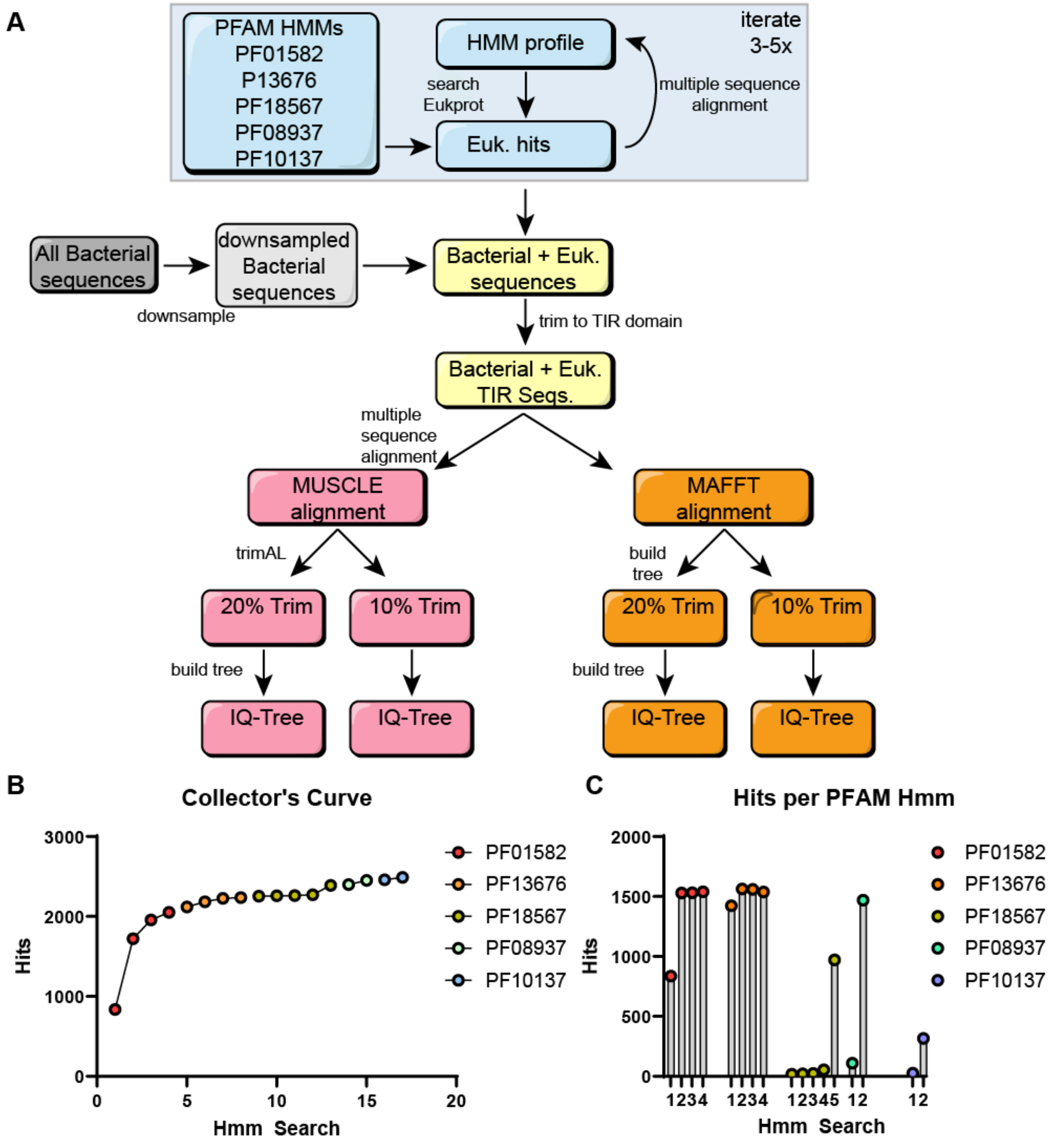

Legend: Supp. Fig. 1: **TIR domain search across multiple PFAM HMMs**
A. Schematic of search strategy employed to find diverse TIR domains across the eukaryotic tree of life. **B.** Collector's curve of searches (left) and **C.** the total number of hits per Pfam Hmm search.

bioRxiv preprint doi: https://doi.org/10.64898/2026.04.30.722052; this version posted May 5, 2026. The copyright holder for this preprint (which was not certified by peer review) is the author/funder, who has granted bioRxiv a license to display the preprint in perpetuity. It is made available under aCC-BY 4.0 International license.

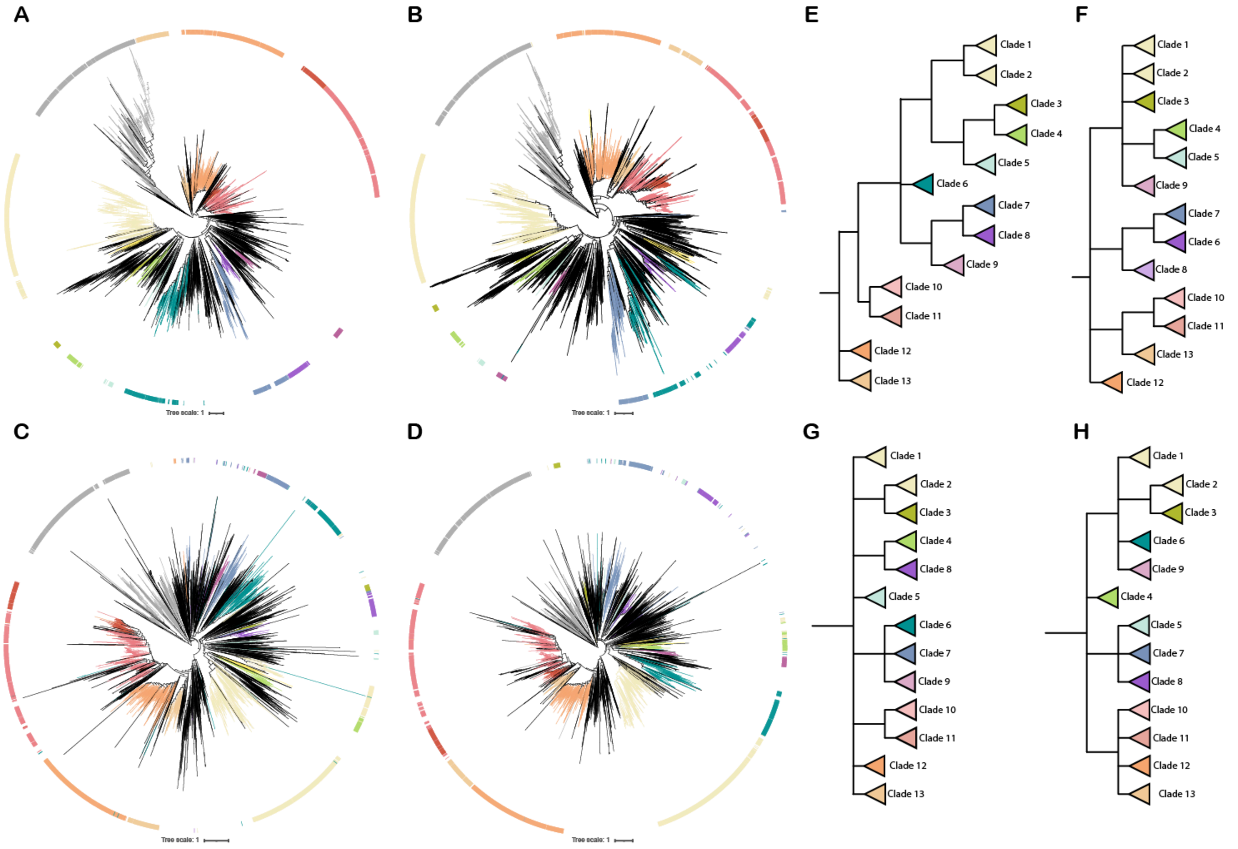

Legend: Supp. Fig. 2: **TIR domain clades are robust across alignment parameters**
Four phylogenetic trees generated by IQtree from varied alignments, showing clades supported across all trees and a schematic of the clade topologies. **A.** Alignment generated via MUSCLE with a 20% trim. **B.** Alignment generated via MUSCLE with a 10% trim. **C.** Alignment generated via MAFFT with a 20% trim. **D.** Alignment generated via Mafft with a 10% trim. **E-H,** Collapsed clades from the four IQtrees (**E**-Muscle 20% trim, **F**-Muscle 10% trim, **G**-MAFFT 20% trim, **H**-MAFFT 10% trim) with clades colored as in Fig. 1. Only nodes with 50 or greater bootstrap values are shown, unsupported nodes are collapsed to polytomies.

bioRxiv preprint doi: https://doi.org/10.64898/2026.04.30.722052; this version posted May 5, 2026. The copyright holder for this preprint (which was not certified by peer review) is the author/funder, who has granted bioRxiv a license to display the preprint in perpetuity. It is made available under aCC-BY 4.0 International license.

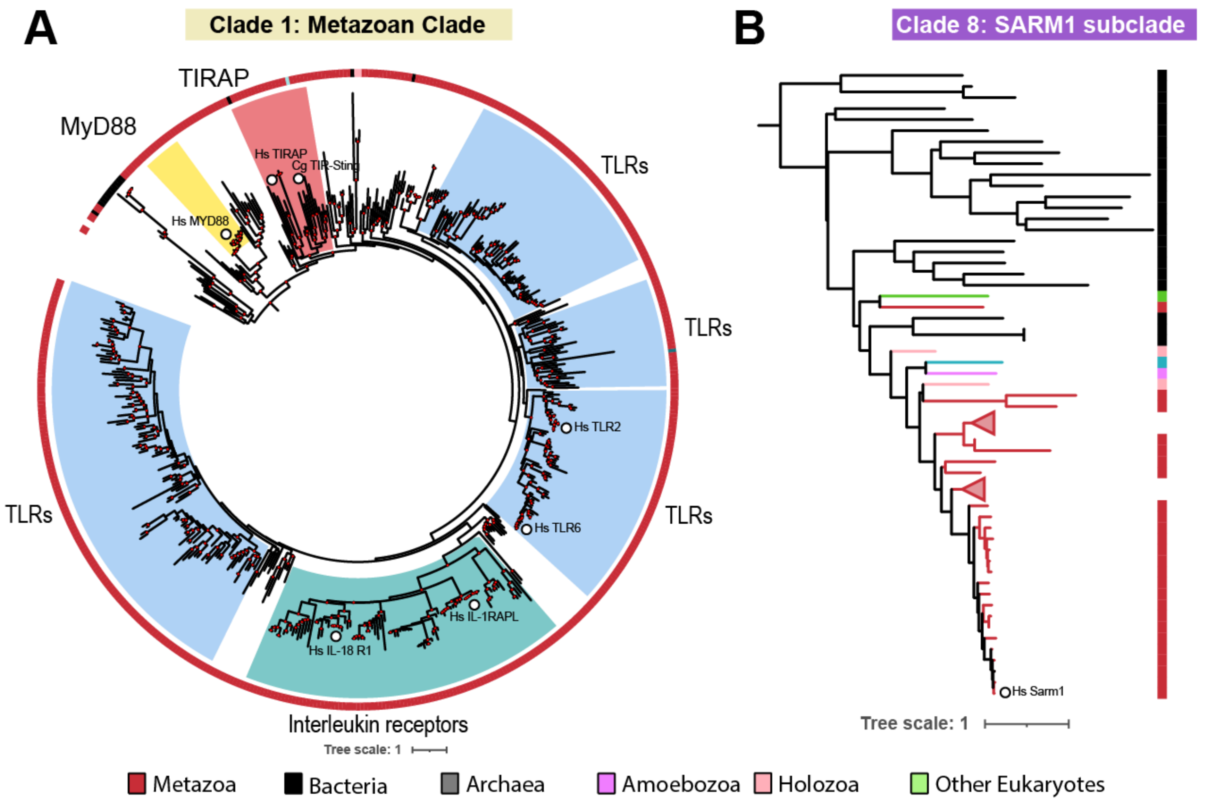

Legend: Supp. Fig. 3: **TIRs family members in clades 1 & 4**

A. Maximum likelihood phylogenetic tree generated by IQtree of TIR domains from Clade 1. **B.** A zoom-in of the Metazoan clade of Clade 1, displayed as a circular tree. The outer ring color indicates the eukaryotic supergroup. **C.** Subset of the maximum likelihood phylogenetic tree of Clade 4.

bioRxiv preprint doi: https://doi.org/10.64898/2026.04.30.722052; this version posted May 5, 2026. The copyright holder for this preprint (which was not certified by peer review) is the author/funder, who has granted bioRxiv a license to display the preprint in perpetuity. It is made available under aCC-BY 4.0 International license.

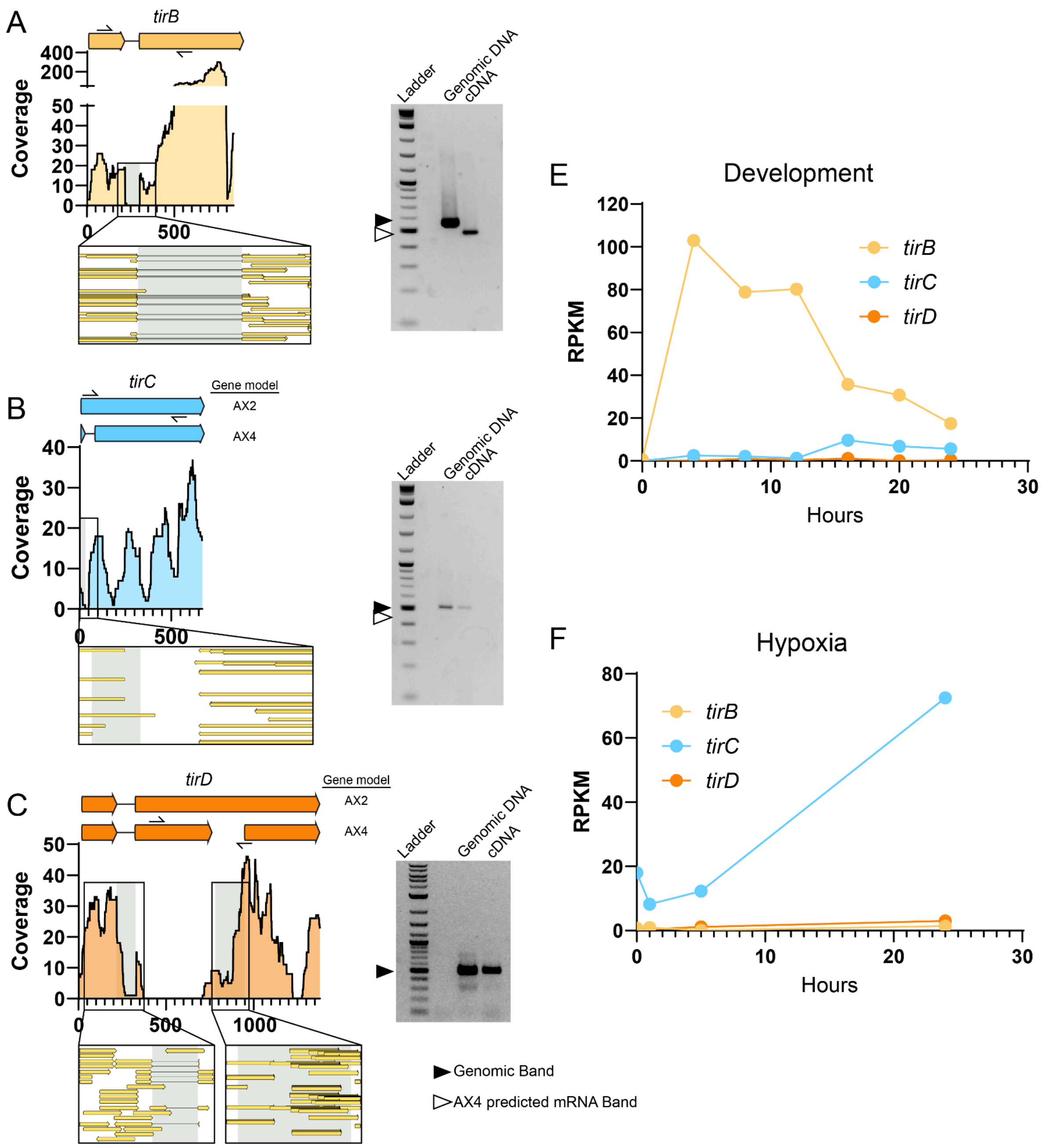

Legend: Supp. Fig 4: ***tirBCD* gene models**

A-C. Diagrams of predicted gene models for *tirB* (**A**), *tirC* (**B**), and *tirD* (**C**). For each gene, the AX2 and AX4 annotations are shown to scale. For *TirB* the two annotations agree and so a single schematic of the coding sequence is shown. For *TirD*, the AX4 annotation predicted that the gene was two genes (*DDB_G028737* and *DDB_G0287321*). Below each schematic is a plot showing the coverage via RNAseq at each position on the genomic locus. The focus box zooms in on the indicated area to show the alignment of individual reads to the genomic locus. The gray boxes indicate the predicted introns by both AX2 and AX4 and are displayed on both the coverage plots and the focus boxes. To the right of the RNAseq cover plots is an Agarose gel

bioRxiv preprint doi: https://doi.org/10.64898/2026.04.30.722052; this version posted May 5, 2026. The copyright holder for this preprint (which was not certified by peer review) is the author/funder, who has granted bioRxiv a license to display the preprint in perpetuity. It is made available under aCC-BY 4.0 International license.

picture where the PCR products of the primers indicated on the gene locus schematics were used to amplify gDNA or cDNA. The black arrow indicates the expected product size when amplifying genomic DNA while the empty arrow shows the product size of amplified cDNA based on the AX4 genome annotation. For *TirD*, the reverse primer spans the AX4-predicted intron-exon boundary and so no product is predicted. **E.** Reads per Kilobase of transcript per Million reads mapped (RPKM) of *TirB, TirC,* and *TirD* (*DDB_G0287321*) from Parikh et. al. [47] over 24 hours of development on a filter. **F.** Reads per Kilobase of transcript per Million reads mapped (RPKM) of *TirB, TirC,* and *TirD* (*DDB_G0287321*) from Hesnard et. al. [46] over 24 hours of hypoxia exposure.

bioRxiv preprint doi: https://doi.org/10.64898/2026.04.30.722052; this version posted May 5, 2026. The copyright holder for this preprint (which was not certified by peer review) is the author/funder, who has granted bioRxiv a license to display the preprint in perpetuity. It is made available under aCC-BY 4.0 International license.

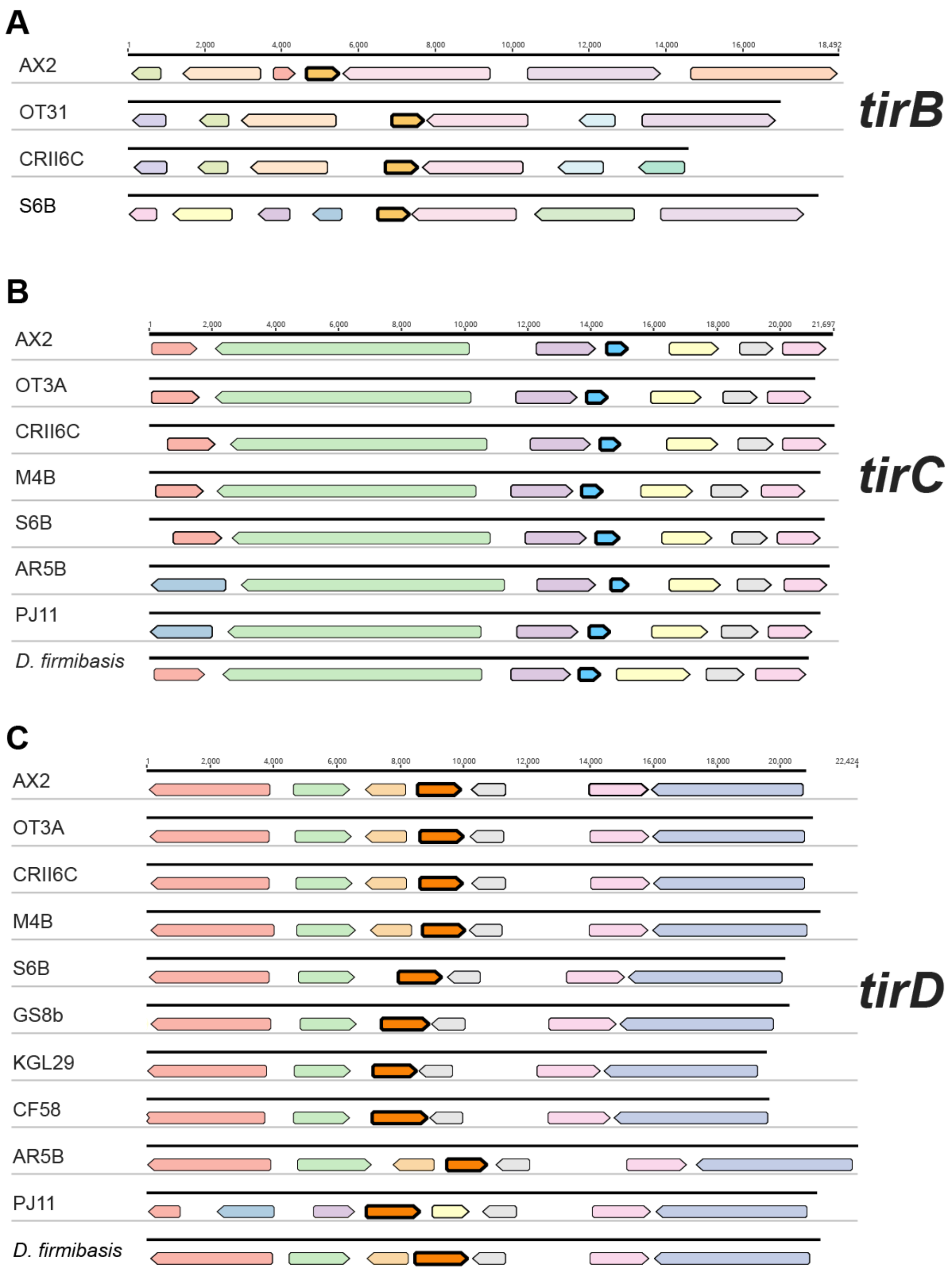

Legend: Supp. Fig 5: **Syntenic distribution of genes at TirB, TirC, TirD genomic loci.** Genetic synteny across Dicyostelium species at the (**A**) TirB, (**B**) TirC, and (**C**) TirD loci. Colored arrows represent homologous genes across the genomes, with strain identifiers listed on the left side. Black outlined arrow represents the TIR-domain containing gene. Numbers above the loci show the nucleotide scale of the locus and genes.

bioRxiv preprint doi: https://doi.org/10.64898/2026.04.30.722052; this version posted May 5, 2026. The copyright holder for this preprint (which was not certified by peer review) is the author/funder, who has granted bioRxiv a license to display the preprint in perpetuity. It is made available under aCC-BY 4.0 International license.

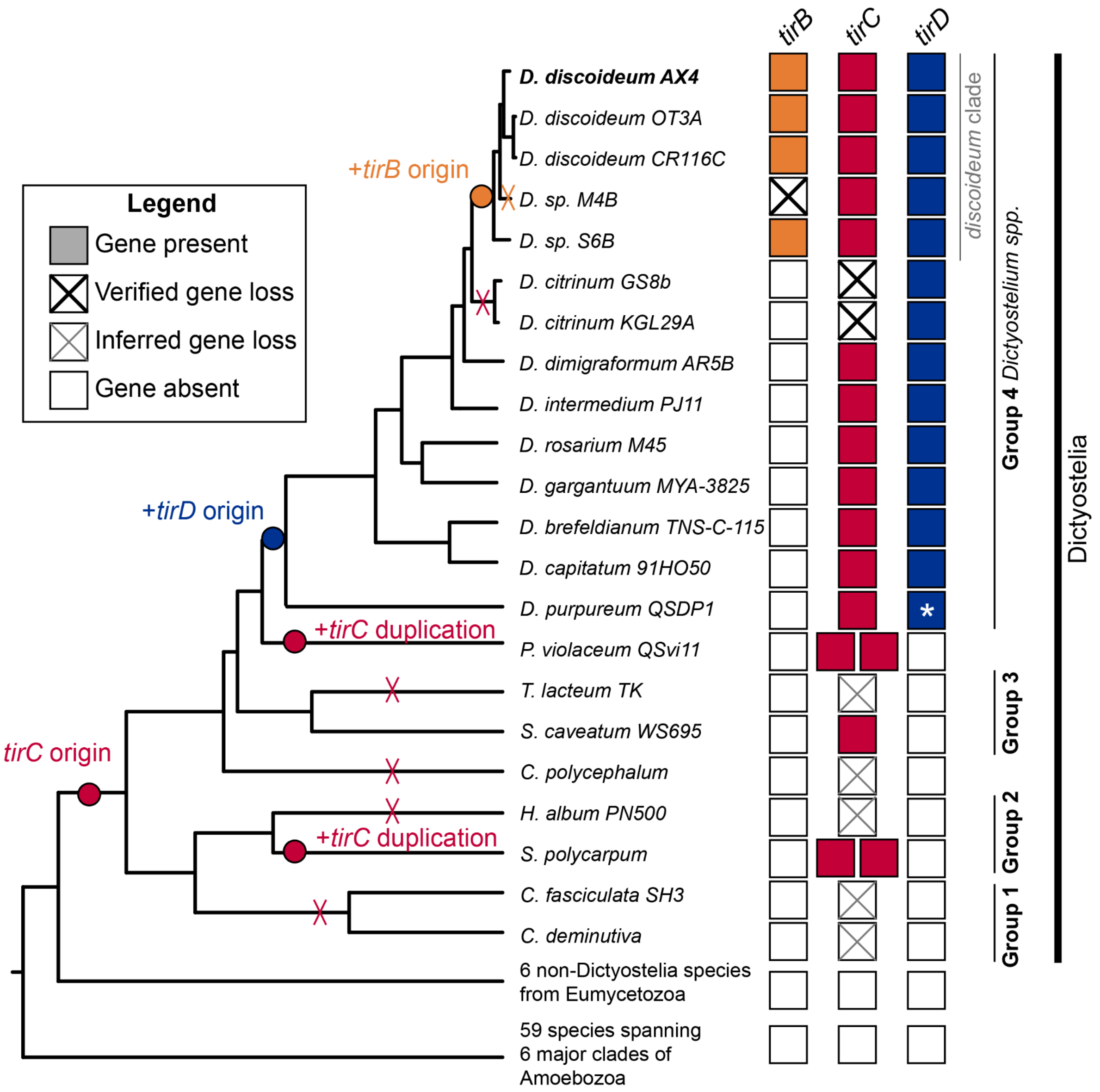

Legend: Supp. Fig 6: **Origin and duplications of *tirBCD* genes within Dictyostelia.** Colored boxes indicate species of cellular slime mold amoebae where *tirB*, *tirC*, and *tirD* genes were present, based on tBLASTn and TIR HMM searches of the amoeba genomes. All colored boxes were discovered by tBLASTn or both methods, except for *D. purpureum* TirD (*), which was identified only by a TIR HMM search. Black Xs mark instances of gene loss where we identified either pseudogenes or deletions in the syntenic locus. Gray Xs mark inferred losses based on the phylogeny, although these apparent absences could result from incomplete assemblies. Outgroup Amoebozoa and Eumycetozoa datasets searched included a combination of genomes and transcriptomes within Eukprot v3. Species tree is based on Schilde et. al. [48], branch lengths not to scale.

bioRxiv preprint doi: https://doi.org/10.64898/2026.04.30.722052; this version posted May 5, 2026. The copyright holder for this preprint (which was not certified by peer review) is the author/funder, who has granted bioRxiv a license to display the preprint in perpetuity. It is made available under aCC-BY 4.0 International license.

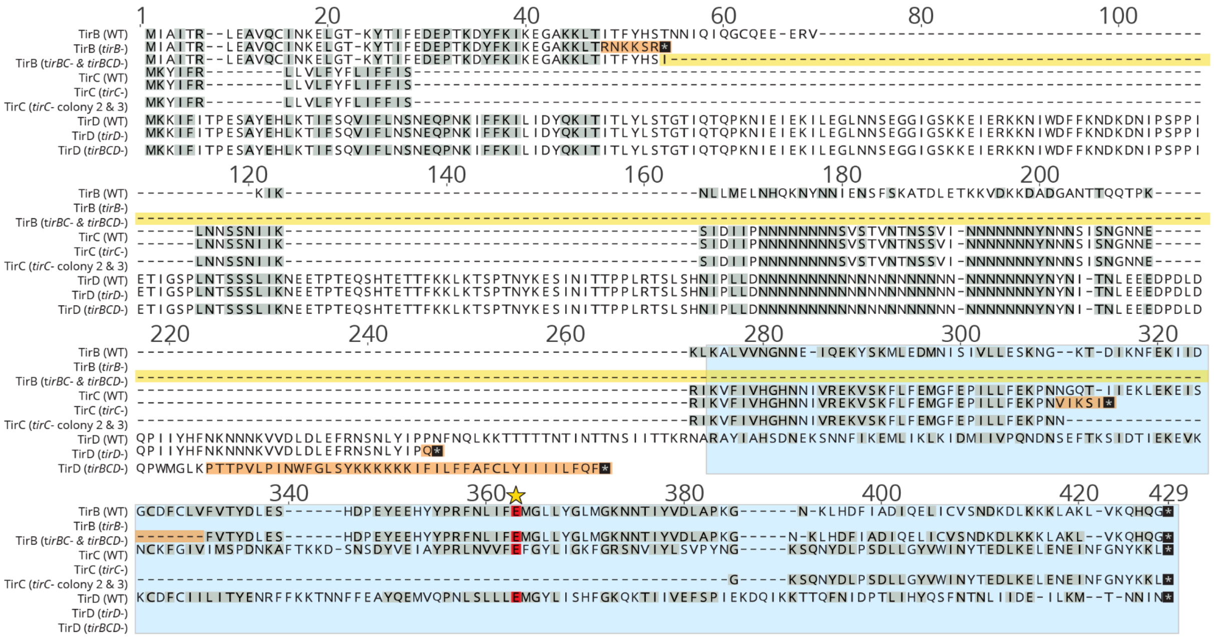

Legend: Supp. Fig. 7: **Amino acid alignment of TirB, TirC, and TirD and their mutants.** Amino acid alignment of wild type TirB, TirC, and TirD and the predicted protein products made by genetically modified strains. The blue box indicates the TIR domain of the proteins. The orange boxes indicate frameshift induced mutations in the protein. The yellow box highlights the amino acids of the protein that have been deleted. Stop codons are highlighted with a black box. The catalytic glutamate is designated with a red box and the locus is marked with a yellow star.

bioRxiv preprint doi: https://doi.org/10.64898/2026.04.30.722052; this version posted May 5, 2026. The copyright holder for this preprint (which was not certified by peer review) is the author/funder, who has granted bioRxiv a license to display the preprint in perpetuity. It is made available under aCC-BY 4.0 International license.

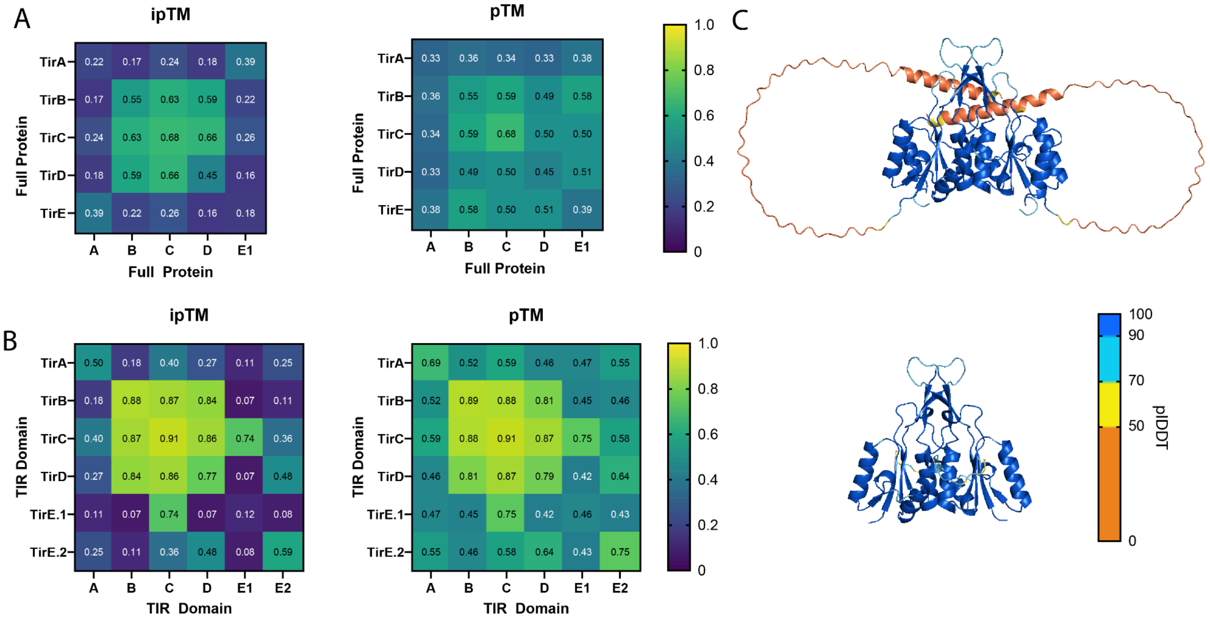

Legend: Fig. 8: **TirC is predicted to homodimerize**

A. ipTM and pTM scores of AlphaFold Multimer predictions of *D. discoideum* TIR full protein dimers. **B.** ipTM and pTM scores of AlphaFold Multimer predictions of *D. discoideum* TIR domain dimers.**C.** AlphaFold models of full length (top) and TIR domain (bottom) TirC homodimers. Residues are colored by pIDDT score.

bioRxiv preprint doi: https://doi.org/10.64898/2026.04.30.722052; this version posted May 5, 2026. The copyright holder for this preprint (which was not certified by peer review) is the author/funder, who has granted bioRxiv a license to display the preprint in perpetuity. It is made available under aCC-BY 4.0 International license.

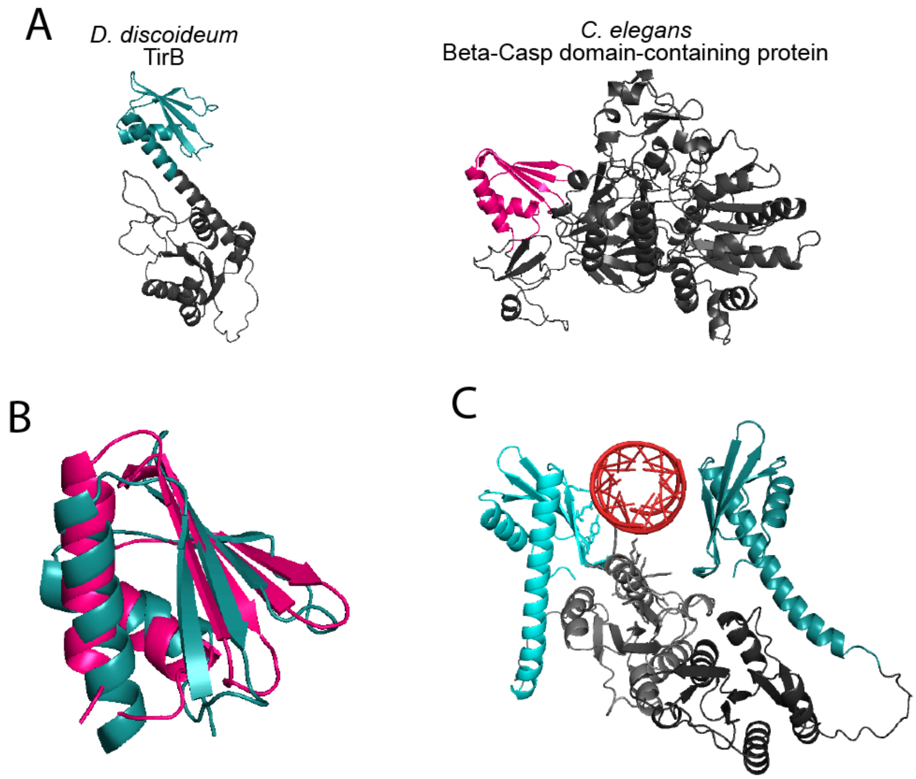

Legend: Fig. 9: **Predicted RNA binding in TirB's N-terminus**
A. AlphaFold predictions of *D. discoideum* TirB and *C. elegans* Beta-Casp domain-containing protein, the highest scoring FoldSeek hit for TirB's N-terminus. The similar domains are colored, teal in TirB, and pink partial KH domain. **B.** Structural alignment of the KH domain of *C. elegans* Beta-Casp domain-containing protein and TirB's N-terminus. The two structures aligned with an RMSD of 3.8 over 45 atoms. **C.** AlphaFold models of full length TirB-TirB homodimers binding a dsRNA molecule. dsRNA is shown in red, while the N-Termini of TirB are shown in shades of teal, and the TIR domain in gray. TirBs are differentiated by shading (protomer 1 is light, protomer 2 is dark).

bioRxiv preprint doi: https://doi.org/10.64898/2026.04.30.722052; this version posted May 5, 2026. The copyright holder for this preprint (which was not certified by peer review) is the author/funder, who has granted bioRxiv a license to display the preprint in perpetuity. It is made available under aCC-BY 4.0 International license.

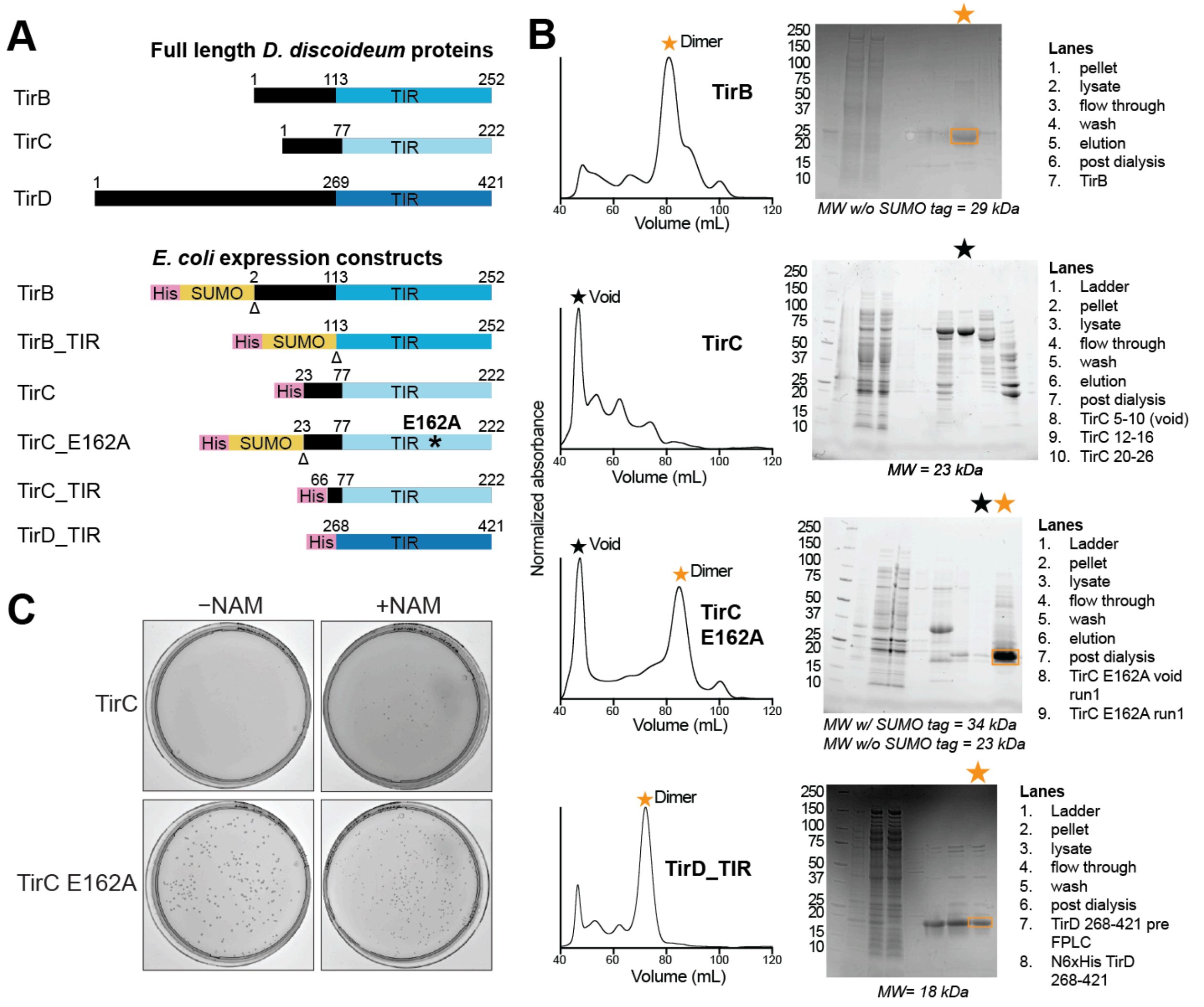

Legend: Supp. Fig. 10: **Purification of TirB, TirC, and TirD.**

A. The SUMO2 tag was proteolytically removed before experimentation. His-tagged proteins had GS linkers between the tag and the protein. Numbers indicate the amino acid coordinates of TirB, C, and D proteins that were included. The TirC construct excluded the N-terminal hydrophobic alpha helix to facilitate protein expression and purification. * shows the location of the E162A point mutation. Δ indicates the SUMO2 cleavage site. **B.** Recombinant protein was purified via size exclusion chromatography (traces on left) and purity of fractions were determined by SDS-PAGE (gel images on right). Lanes are numbered from left to right. Stars indicate primary fractions considered for activity assays. Note that for WT TirC there was no obvious band on the gel and yet the void fraction contains measurable NADase activity (as shown in other figures). Contaminants near 20 kDa and 75 kDa are common E. coli proteins which bind nickel-resin with appreciable affinity under the conditions tested. **C.** TirC is toxic to E. coli, but partially rescued by extracellular nicotinamide. Images show colonies of bacteria on MDG agar plates in the absence of inducer, with toxicity due to leaky TirC expression from the T7 promoter. The E162A TirC mutant construct was not toxic.

bioRxiv preprint doi: https://doi.org/10.64898/2026.04.30.722052; this version posted May 5, 2026. The copyright holder for this preprint (which was not certified by peer review) is the author/funder, who has granted bioRxiv a license to display the preprint in perpetuity. It is made available under aCC-BY 4.0 International license.

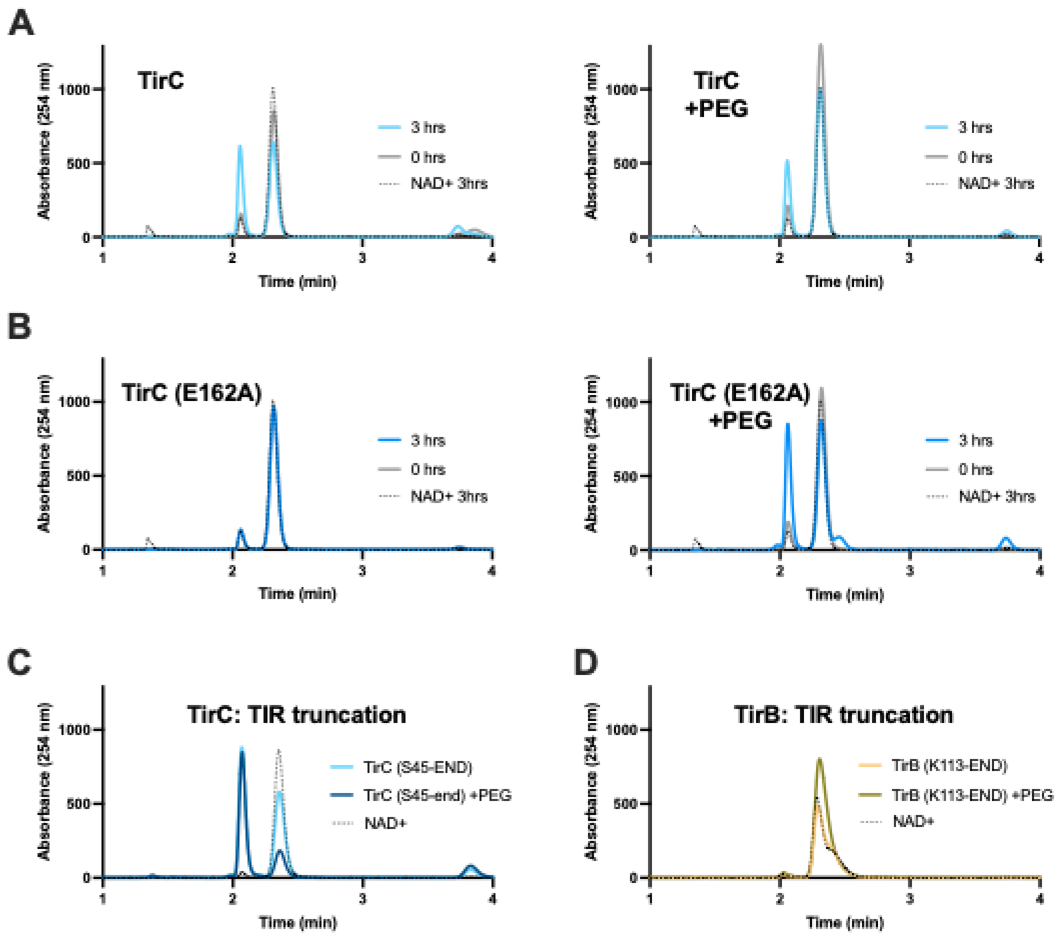

Legend: Supp. Fig. 11: **NAD+ processing by TirC and TirB mutants in the presence of PEG 8000.**

A. Time course of *in vitro* co-incubations of NAD+ and WT TirC in the presence of PEG 8000 as a molecular crowding agent. In both cases, WT TirC cleaves NAD to produce ADPR and NAM. Black dotted lines show the absorbance of NAD+ alone, following 3h incubation in the study conditions. Colored lines show traces after 0-3h co-incubation with protein. **B.** Similar time course using the TirC E162A mutant, which cleaves NAD+ only in the presence of PEG 8000. **C.** TirC truncation mutant (S45-END) that includes only the TIR domain processes NAD+ similar to TirC WT, with only a slight change in processing rate in the presence of PEG. **D.** Truncation mutant of TirB (K113-END) to the TIR domain does not show any NAD+ cleavage activity, even in the presence of PEG.

bioRxiv preprint doi: https://doi.org/10.64898/2026.04.30.722052; this version posted May 5, 2026. The copyright holder for this preprint (which was not certified by peer review) is the author/funder, who has granted bioRxiv a license to display the preprint in perpetuity. It is made available under aCC-BY 4.0 International license.

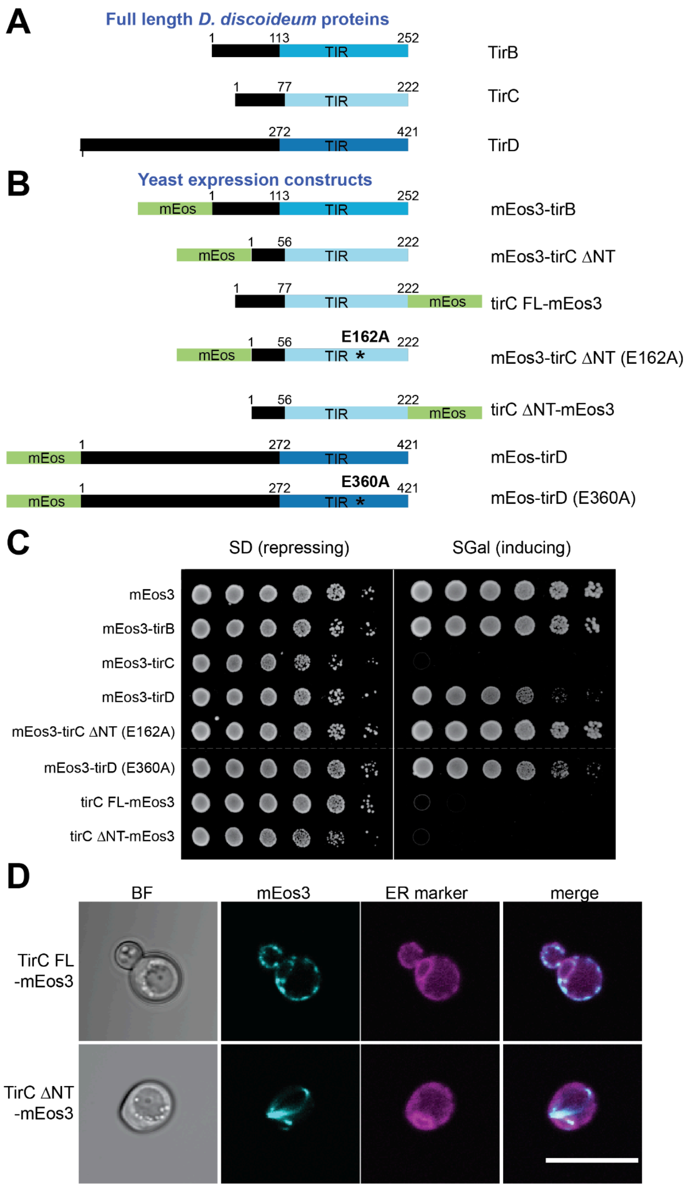

Legend: Supp. Fig. 12: **Expression of TirBCD in yeast**.

A & B. Schematic of *D. discoideum* TIR proteins expressed in *S. cerevisiae*. Numbers indicate the coordinates of TirB, C, and D proteins that were included. FL = full-length, ΔNT = N-terminal helix is missing, * shows the location of the putative catalytic glutamate. To facilitate DAmFRET and protein localization studies, the amoeba proteins were tagged with mEos fluorophores. **C**. Viability assay of yeast carrying the TirBCD expression constructs were serially diluted (5-fold) and spotted onto selective media either to repress (SD) or induce (Sgal) protein expression. **D.** Localization of TirC FL or ΔNT proteins in yeast expressing the mTagBFP2 ER marker. Cells were grown in excess nicotinamide to enhance TirC expression. TirC FL strongly co-localizes with the ER but TirC ΔNT does not, suggesting that the N-terminal helix facilitates membrane localization.

bioRxiv preprint doi: https://doi.org/10.64898/2026.04.30.722052; this version posted May 5, 2026. The copyright holder for this preprint (which was not certified by peer review) is the author/funder, who has granted bioRxiv a license to display the preprint in perpetuity. It is made available under aCC-BY 4.0 International license.

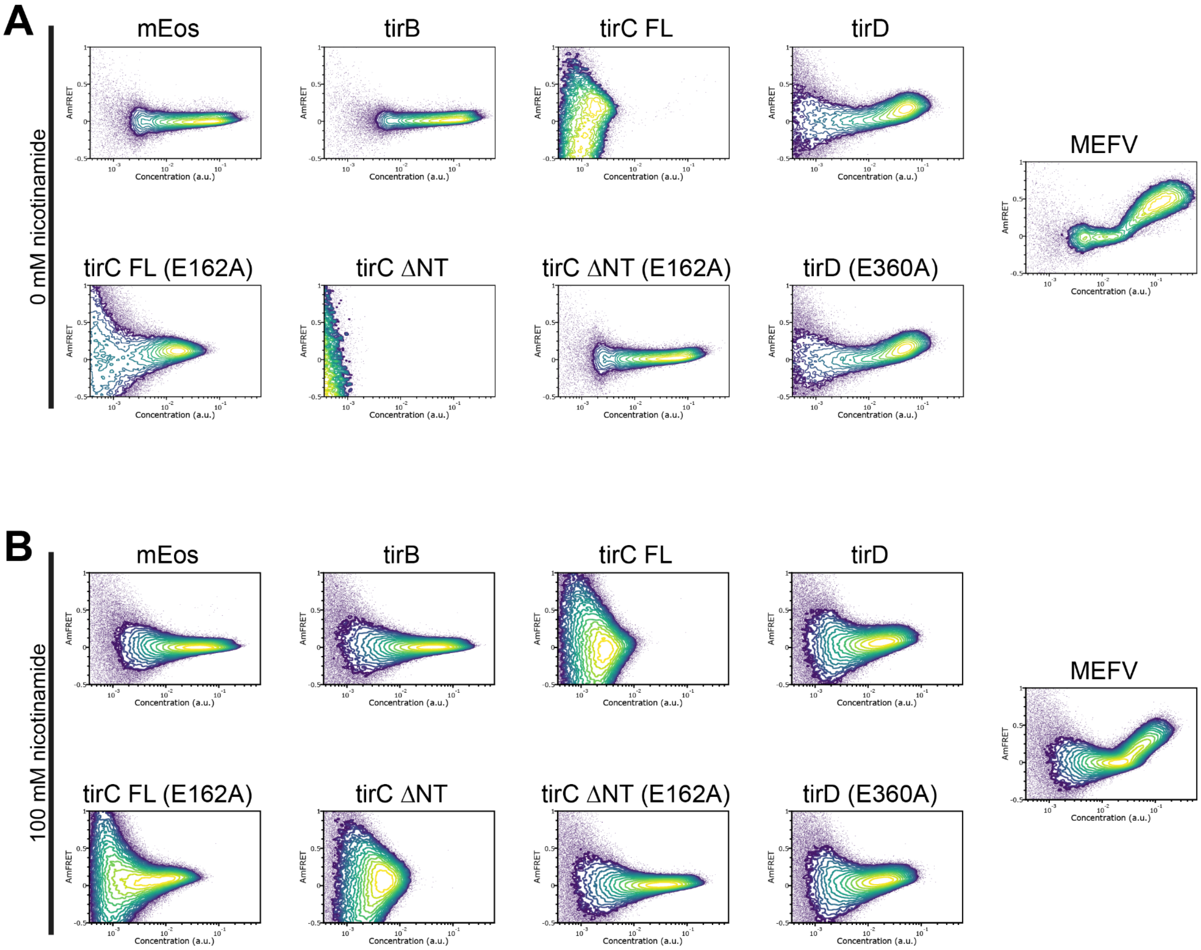

Legend: Supp. Fig. 13: **Self oligomerization of TIR proteins via DAmFRET.** Representative DAmFRET data showing the propensity of various *D. discoideum* TIR constructs to oligomerize in yeast cells. mEos serves as a negative control, which fails to aggregate even at high concentrations, while MEFV is a positive control for aggregation and filamentation. Cells were grown without supplemental nicotinamide (**A**) and with 100 mM nicotinamide (**B**).

bioRxiv preprint doi: https://doi.org/10.64898/2026.04.30.722052; this version posted May 5, 2026. The copyright holder for this preprint (which was not certified by peer review) is the author/funder, who has granted bioRxiv a license to display the preprint in perpetuity. It is made available under aCC-BY 4.0 International license.

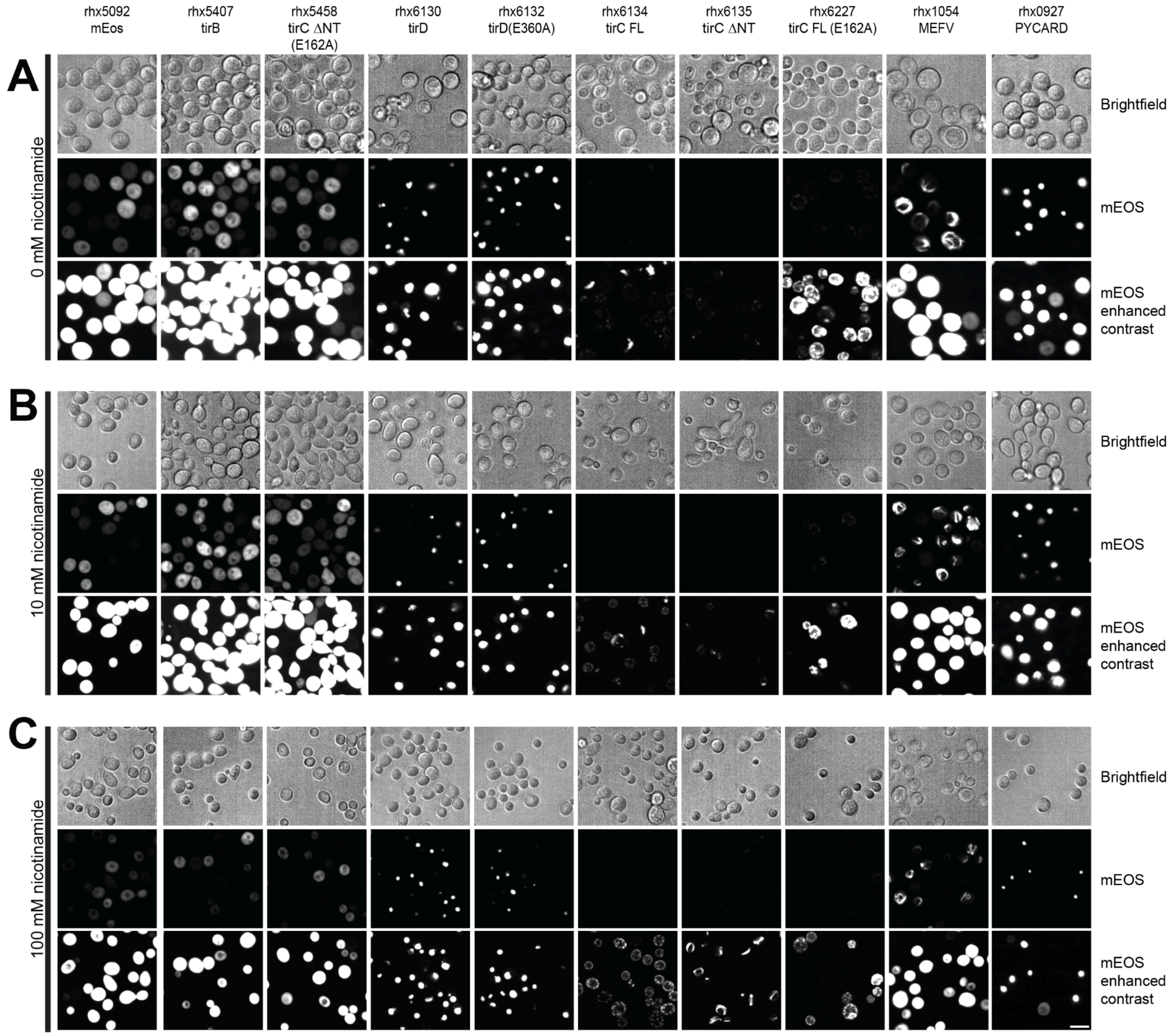

Legend: Supp. Fig. 14: **TIR construct localization in yeast.**

Microcopy images of yeast cells expressing various mEos3-tagged constructs. Images are from brightfield and the green (mEos3) channel. The mEOS channel is displayed twice with the contrast on the second image being enhanced to allow visualization of low mEos3 expression. Yeast cells were grown without supplemental nicotinamide (**A**), with 10 mM nicotinamide (**B**), and with 100 mM nicotinamide (**C**). Scale bar is 10 µm.

bioRxiv preprint doi: https://doi.org/10.64898/2026.04.30.722052; this version posted May 5, 2026. The copyright holder for this preprint (which was not certified by peer review) is the author/funder, who has granted bioRxiv a license to display the preprint in perpetuity. It is made available under aCC-BY 4.0 International license.

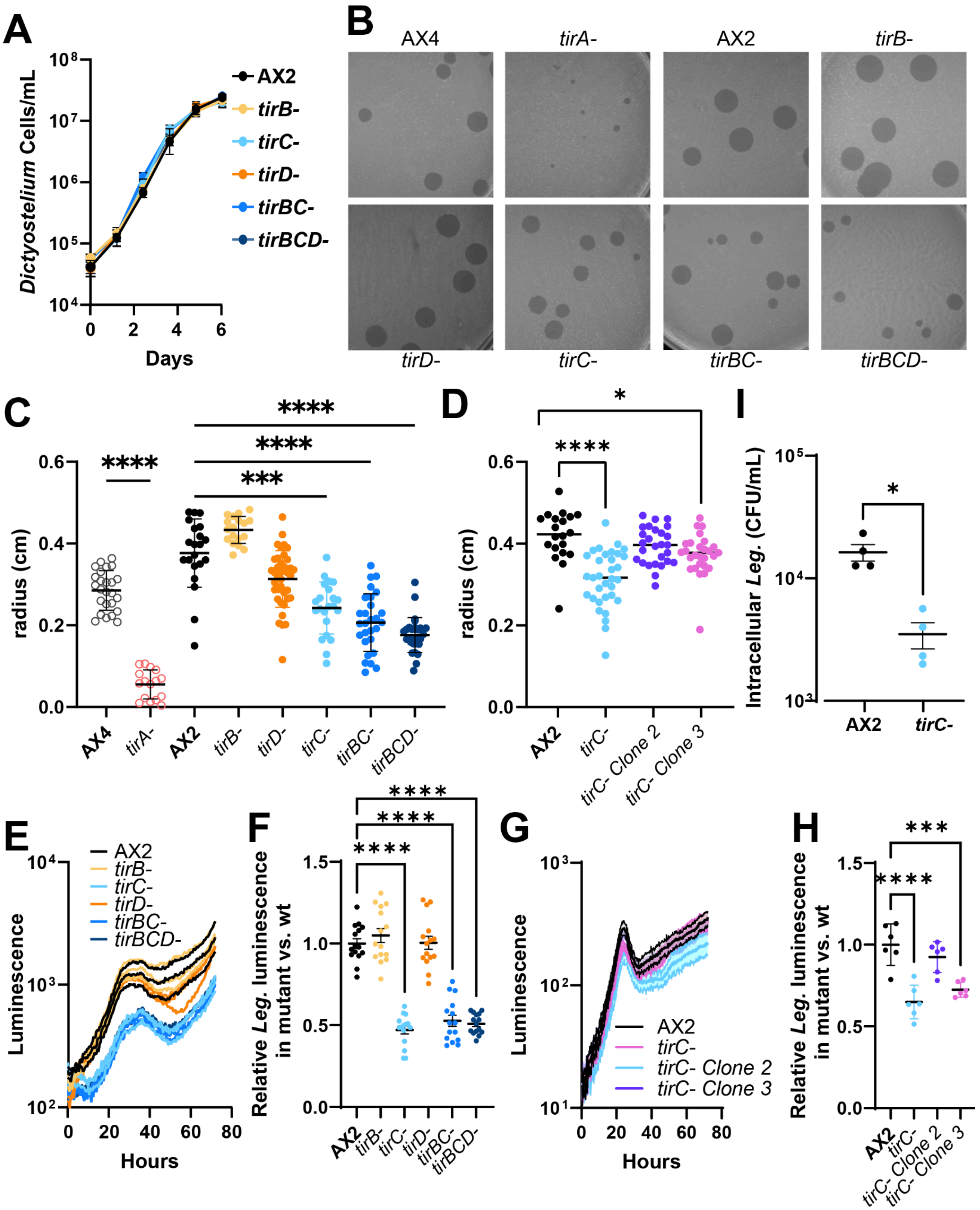

Legend: Supp. Fig. 15: ***tirC-* mutants are defective in bacterial uptake.**

A. Growth curve of wild type (AX2) and TIR mutant *D. discoideum* cultures in HL5 rich media. The TirBCD proteins are not essential for cell viability or growth. **B.** Representative photos of *D.*

bioRxiv preprint doi: https://doi.org/10.64898/2026.04.30.722052; this version posted May 5, 2026. The copyright holder for this preprint (which was not certified by peer review) is the author/funder, who has granted bioRxiv a license to display the preprint in perpetuity. It is made available under aCC-BY 4.0 International license.

discoideum plaque sizes on a lawn of *K. pneumoniae* measured after four days of growth for all *D. discoideum* strains. **C-D.** Quantification of plaques across three replicate plates per genotype, measured after four days. Panel **C** shows plaque size across multiple genotypes while panel **D** shows plaque size variation amongst different tirC- clones. **E,G.** Curves of luminescent Lg during infection of *D. discoideum* TIR mutants over 72 hours. *D. discoideum* cells were infected with Lg at an MOI of 10 and incubated at 22°C for three days. Experiment was carried out in triplicate wells with the luminescence curve of each well plotted. **F,H.** Comparison of the peak luminescence relative to wild-type reached by each strain between the 20-40 hours post infection window. Panel **E-F** shows *Legionella* luminescence across multiple genotypes while panel **G-H** shows variation amongst different *tirC-* clones. **I.** Gentamicin protection assay during *L. pneumophila* infection of AX2 and *tirC-*. Statistical significance for C,D, was determined by one-way ANOVA with Kruskal-Wallis posttest and statistical support for F,H was determined by one-way ANOVA with Kruskal-Wallis posttest; $^*p \leq .05$,$^{***}p \leq .001$, $^{****}p \leq .0001$. Statistical significance for I was determined by two tailed student's t-test; $^*p \leq .05$.

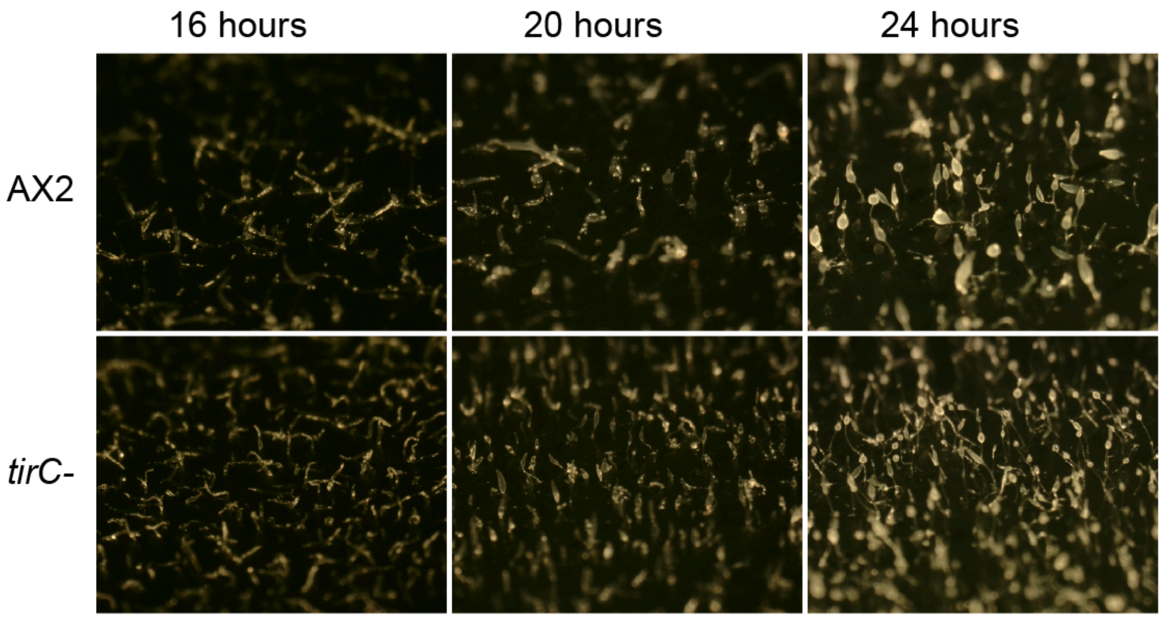

Legend: Supp. Fig. 16: **Development of fruiting bodies.**
Photographs of the development of fruiting bodies in *D. discoideum* wild type (AX2) and *tirC-* over 24 hours.

bioRxiv preprint doi: https://doi.org/10.64898/2026.04.30.722052; this version posted May 5, 2026. The copyright holder for this preprint (which was not certified by peer review) is the author/funder, who has granted bioRxiv a license to display the preprint in perpetuity. It is made available under aCC-BY 4.0 International license.

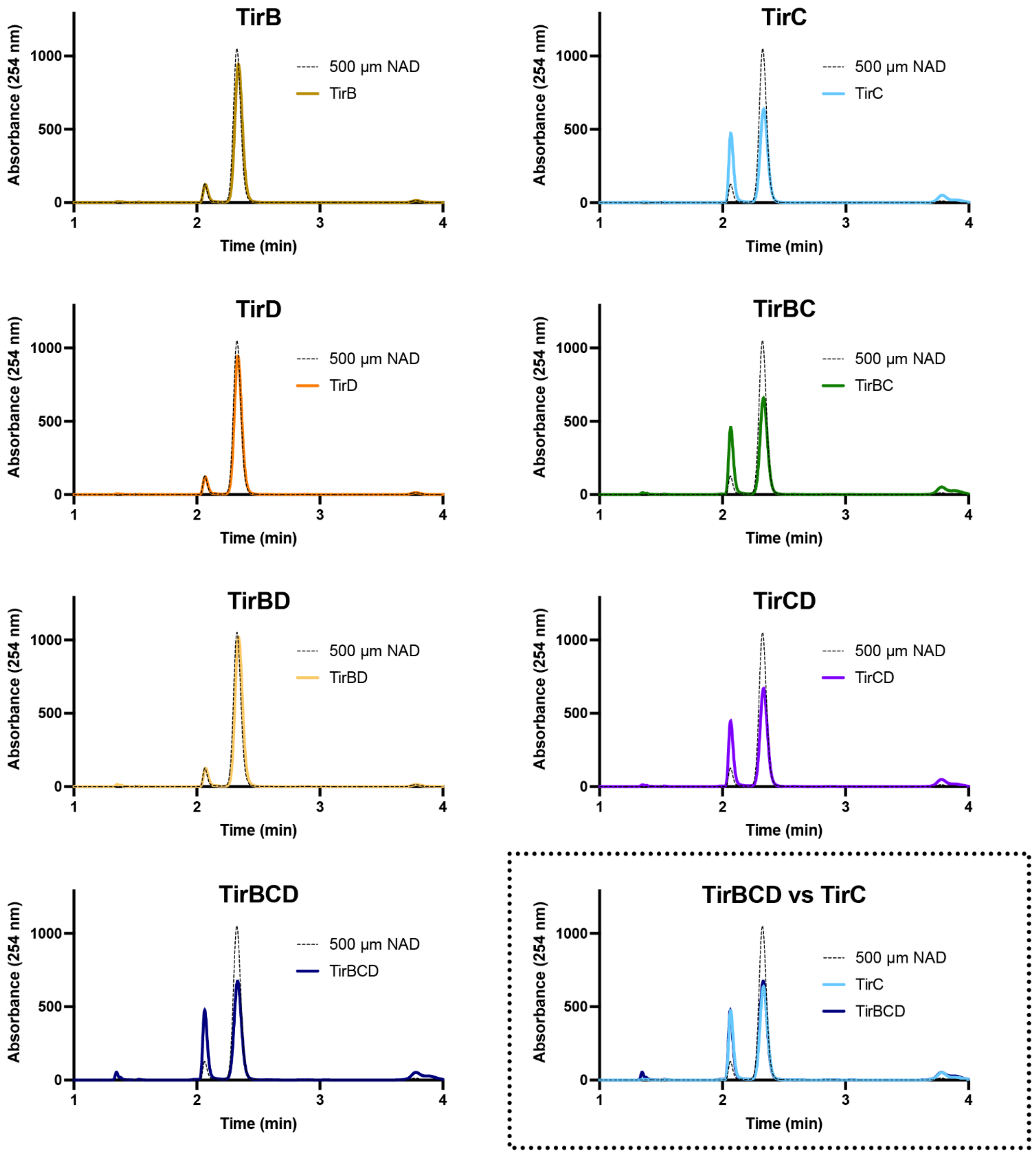

Legend: Supp. Fig. 17: **Co-incubation of TirB, C, and D proteins *in vitro* does not alter NADase activity.**

High-performance liquid chromatography analysis of NAD+ incubated with purified TirB, TirC, TirD, or combinations thereof. We observed no NAD+ processing from TirB or TirD, alone or with each other. In all other mixes, the observed activity was equivalent to the activity of TirC alone. The graph in the dotted box overlays the TirC-only results with those from the TirB, C, and D mix, showing that they are nearly identical.

bioRxiv preprint doi: https://doi.org/10.64898/2026.04.30.722052; this version posted May 5, 2026. The copyright holder for this preprint (which was not certified by peer review) is the author/funder, who has granted bioRxiv a license to display the preprint in perpetuity. It is made available under aCC-BY 4.0 International license.

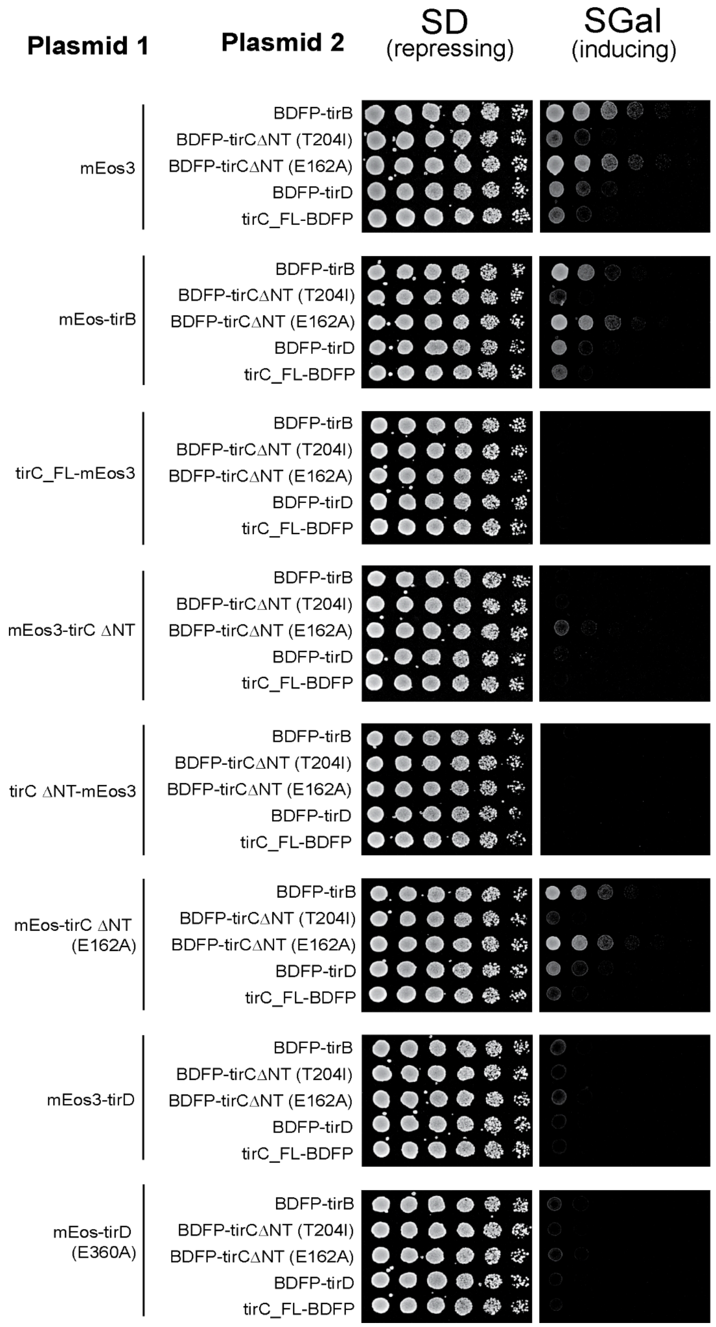

Legend: Supp. Fig. 18: ***Toxicity of TirBCD co-expression in S. cerevisiae.***
Yeast cells carrying the indicated plasmid constructs were serially diluted (5-fold) and spotted onto selective media to either repress or induce co-expression of the indicated mEos- and BDFP-tagged proteins. TirC constructs were either full length (FL) or truncated to remove the N-terminal helix (ΔNT). Plates were incubated at 30°C for 3 days. Images are representative of 2 replicates of the spotting assay.

bioRxiv preprint doi: https://doi.org/10.64898/2026.04.30.722052; this version posted May 5, 2026. The copyright holder for this preprint (which was not certified by peer review) is the author/funder, who has granted bioRxiv a license to display the preprint in perpetuity. It is made available under aCC-BY 4.0 International license.

Supplemental Movie

Legend: Supp. Mov. 1: **D. Discoideum expressing full length WT tirC over 16 hours**
Fluorescent microscopy time lapse of D. discoideum expressing full length WT tirC tagged with mNeon on the C terminus. The time lapse covers 16 hours with a 10 minute frame interval. The time stamp in the top right corner displays times in an hour:minute format.
